# Recent Advancements in Gel Polymer Electrolytes for Flexible Energy Storage Applications

**DOI:** 10.3390/polym16172506

**Published:** 2024-09-03

**Authors:** Thi Khanh Ly Nguyen, Thuan-Nguyen Pham-Truong

**Affiliations:** Laboratory of Physical Chemistry of Polymers and Interfaces (LPPI), CY Cergy Paris Université, F-95000 Cergy, France

**Keywords:** gel polymer electrolytes, hydrogels, organogels, ionogels, mechanical deformation, self-healing, batteries, supercapacitors

## Abstract

Since the last decade, the need for deformable electronics exponentially increased, requiring adaptive energy storage systems, especially batteries and supercapacitors. Thus, the conception and elaboration of new deformable electrolytes becomes more crucial than ever. Among diverse materials, gel polymer electrolytes (hydrogels, organogels, and ionogels) remain the most studied thanks to the ability to tune the physicochemical and mechanical properties by changing the nature of the precursors, the type of interactions, and the formulation. Nevertheless, the exploitation of this category of electrolyte as a possible commercial product is still restrained, due to different issues related to the nature of the gels (ionic conductivity, evaporation of filling solvent, toxicity, etc.). Therefore, this review aims to resume different strategies to tailor the properties of the gel polymer electrolytes as well as to provide recent advancements in the field toward the elaboration of deformable batteries and supercapacitors.

## 1. Introduction

With the increasing demand for foldable, flexible, and wearable electronics to respond to the emerging era of Internet-of-Things (IoTs), the development of highly efficient, flexible, and deformable energy supply systems is becoming one of the keys to unlocking the realm of these electronic devices. At the current state, batteries and supercapacitors are two main categories of energy storage systems that could be suitable for this purpose, working mainly on three distinct mechanisms: the electrostatic adsorption of ions (EDLC mechanism), the non-capacitive Faradaic or Nernstian mechanism, and the pseudocapacitive charge storage mechanism. A combination of these storage pathways leads to different types of electrochemical storage devices, including batteries [1,2,3], EDL capacitors [4], pseudocapacitors [5], hybrid electrochemical capacitors [6], and supercabatteries [7]. Depending on the type of system, they can provide high energy. high power or both. Nevertheless, independent of their names and operation mechanisms, these systems share the same cell configuration, including an electrolyte (and/or polymeric separator) in a sandwich between two electrodes (positrode and negatrode). While a large library of active electrode materials has been significantly developed, the electrolytes have remained humbler in their developments with the traditional use of salts dissolved in solvent (water, acetonitrile, carbonate-based organic solvents, etc.), or ionic liquid and deep eutectic solvents [8]. Despite developing high-performing systems, they will never be applied in such devices as liquid electrolytes cannot be free-standing, and under mechanical constraints, the liquid electrolyte will generate enormous pressure on the encapsulation layer, increasing the risk of the cell’s burst. Moreover, they face major issues for deformable/wearable devices due to the low operation voltage (water), the risk of electrolyte leakage, inflammability (organic solvents), and toxicity (ionic liquid/deep eutectic/organic solvents). In light of this situation, all-solid-state or quasi-solid-state electrolytes are required to have electrochemical storage systems that have appropriate safety and mechanical properties. Thus, freestanding polymer electrolytes allow them to bring excellent flexibility, compressibility, stretchability, and self-healing properties to energy storage systems. However, imprisoning ions within a solid structure reduces their mobility and thus, reduces the ionic conductivity of the electrolyte (several orders of magnitude) [9]. Therefore, the above gains are counter-balanced to the detriment of the electrochemical performance. Among candidates, gel polymer electrolytes (GPEs: ionogel, hydrogels, organogels) emerge as promising candidates to ensure the mechanical properties of the devices while allowing the ions to circulate within their structure [10]. Typically, one or more polymer networks are adopted in the GPEs as a host matrix for electrolytes, providing high mechanical integrity. Indeed, a high-performant GPE must satisfy specific requirements for both polymer framework and electrolyte. Concerning polymer host, the key requirements rely on (1) local relaxation and segmental motion of polymer chains [11]; (2) the presence of functional groups promoting dissociation of salts [12]; (3) low glass transition temperature [13]; (4) high thermal stability, and (5) wide electrochemical window to increase the energy density. Concerning the guest electrolytes/solvents, they need to have [14,15,16]: (1) thermal/electrochemical stability, (2) high dielectric constant, (3) low vapor phase pression to prevent drying of the GPEs, (4) salts with large anions and low ion pair dissociation energy barrier to prevent the formation of ion aggregation, yielding high ionic conductivity. In addition to the aforementioned criteria, other dimensions, such as mechanical constraints and thermoresponsibility, need to be included in the conception of good GPE for flexible, stretchable, deformable devices.

Flexibility of electrolytes is defined as the ability of materials to resist repeated mechanical deformation such as bending, distorting, folding, stretching, or compressing without alternating their electrochemical performance [17,18], which permits the whole system to work correctly under a variety of conditions. Stretchability/compressibility is more difficult to achieve since it requires the reversibility of the original form and characteristics of electrolytes after deformation [19]. The change in the dimension of material under externally applied force (strain) is often used as a measured parameter for flexibility or stretchability. However, different from conventional mechanical tests, it is important to note the maximum strain that the electrolytes can sustain without changing their characteristics [20]. Three principles ways to acquire flexible/stretchable electrolytes consist of (i) elaborating inherently stretchable conducting materials [21], (ii) introducing external fillers to reinforce the system’s elastomeric/rigidity [22], and (iii) developing elastic patterns as support for electrolytes integrating [23]. As an example, a newly developed stretchable gel polymer matrix from poly (ethylene oxide) (PEO)-poly (propylene oxide) (PPO)-poly (ethylene oxide) (Pluronic) in 1 M KCl aqueous electrolyte can sustain up to 1535% strain with remarkable room temperature conductivity (27.57 mS cm^−1^) [21].

Self-healing is a promising approach to introduce the self-healing materials flexible electrolytes in order to intrinsically reverse their properties in case of damage or function failure under deformation [20]. In general, the self-healing process involves four steps, including damage (or crack), triggering, transport of healing precursors, and chemical repair. From the literature, self-healing materials can be differently classified depending on the conductivity of the materials [24] or on the healing mechanism [25]. In the former approach, the self-healing materials can be classified as insulators, electrical conductors, ionic conductors, and semiconductors while in the second approach, the self-healing is distinguished by extrinsic [26,27] or intrinsic [28] mechanism. Up to date, the majority of self-healing polymer networks for batteries and supercapacitors are based on intrinsic mechanisms in which reversible chemical bonds or supramolecular interactions are dominant. The supramolecular interactions with hydrogen bonding and electrostatic interactions as typical examples consist of using directional and non-covalent interactions as major forces to govern the mechanical behaviors of the materials. Accordingly, this type of interaction appears as an ideal candidate for generating intrinsic self-healing polymers since no external stimuli (heat, UV, catalyst) are required. With the pioneered work from Meijer’s group [29] on using quadruple hydrogen bonding from 2-ureido−4-pyrimidone to generate self-assembling polymer networks, many works have been reported on a similar approach [30,31,32,33,34,35]. Interestingly, Huang et al., 2018 [32] have demonstrated the dominance of the ionic self-healing mechanism against hydrogen bonding in ferric ion crosslinking sodium polyacrylate hydrogel electrolytes. Excellent flexibility of healed gel (tensile strain around 1000%) was only obtained with the presence of Fe^3+^ ion in the crosslinked system indicating a completing healing effect. Dynamic covalent bonds, e.g., Diels–Alder [36,37,38], disulfide [39,40], or imine [41,42,43] are more advantageous in terms of infinite healing times, mechanical toughness, and stability [44]. As an example, stretchable ionogel-based dynamic imide crosslinks between NH_2_–PEG–NH_2_ and 1,3,5-benzenetricarboxaldehyde prepared by Wan et al., 2022 [41] are able to maintain their flexibility (strain at break of 600%) after 1 h healing at 25 °C.

Thermoresponsibility. Concerning the security of energy storage systems, thermal runaway could be avoided by making more stable and safer systems versus instant change in temperature through the development of flame retardant or thermally responsive electrolytes. The flame retardant effect can be issued from the intrinsically fireproof polymer matrix [41,45], organic solvent [46,47], or ionic liquids [33] in the gel electrolytes or from external inorganic fillers [48]. However, the use of flame-retardant additives can affect the properties of final gel electrolytes [49] in terms of ionic conduction (high content of fillers) or flexibility (high amount of solvent or ILs). Moreover, these approaches are not able to cease the increase in temperature and flame-retardant mechanisms can also actuate the initial compositions of electrolytes in some cases preventing the reusability of the devices [50]. Thermal-responsive electrolytes become a real alternative allowing the halt of thermal runaway. Different mechanisms of thermally responsive polymers for electrolytes were discussed in the literature including phase change [51,52,53], sol–gel transition [45,54,55,56,57,58], and thermal polymerization [59]. Sol–gel mechanism with thermal switching behavior was the most studied in gel electrolytes thanks to the intelligence and reversibility [49]. In a study of Xu’s group, the thermal association of poly(N-isopropylacrylamide-co-N-methylolacrylamide) chain as a function of increasing temperature to form a gel network hinders conduction paths allowing self-protective mechanism for hydrogel electrolytes [60].

**Scheme 1:**
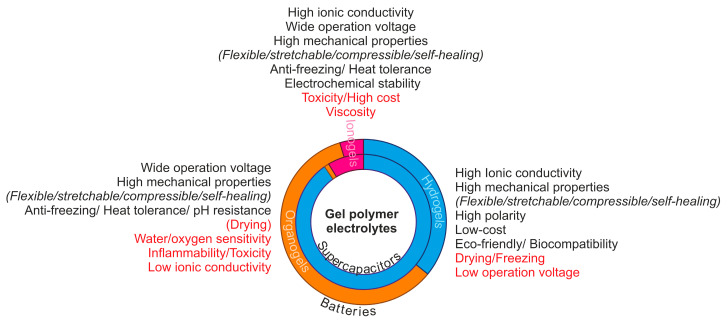
Different types of gel polymer electrolytes and their key features.

It is noteworthy that at the state-of-the-art, a dilemma between the mechanical properties and electrochemical performance is usually observed for all of the reported systems. Therefore, finding the best compromise is still the major challenge in the field of rendering a GPE that satisfies all the requirements. Up-to-date, three major families of GPEs, including ionogels, hydrogels, and organogels are commonly adopted and developed (Scheme 1).

## 2. Design and Applications of GPE in Batteries and Supercapacitors

### 2.1. Ionic Liquid-Based Polymer Gel Electrolytes (IL-GPEs)

This class of GPEs, known as solidified ionic liquid or ionogel, is a result of an incorporation of ionic liquid within a host polymer matrix. Since the first reports of ionogel from 2001–2005 by Nakashima [61], Watanabe [62], and Vioux [63] groups, independently, wide attention has been attributed to the development of this class of gelled electrolytes for electrochemical applications, including energy-related systems, electrochemical/biosensors, catalysis, actuators, biomedical applications, etc. Thanks to their electrochemical and thermal stability, ionic liquids have been largely used to replace conventional solvents as plasticizers for gel polymer electrolytes. Indeed, ionic liquids could provide different types of interactions including, hydrogen bonding [64], electrostatic [65], and host–guest [66] interactions. Due to their compositions, a majority of the ionogels have the marriage of properties between organic and aqueous gel electrolytes with high ionic conductivity (at 298 K) in the mS cm^−1^ range [67] and large electrochemical windows above 5.5 V [68,69]. Moreover, the use of ionogel prevents risks of leakage as well as the deterioration in electrochemical storage performances due to the evaporation of water or organic solvents. All ionogels can be classified into two main categories depending on the nature of immobilized host matrices (organic or inorganic) which can again be classified into sub-classes depending on the interaction of the ionic liquid moieties and the host network (covalent bonding or physical blend or both).

#### 2.1.1. Ionogels for Battery Applications

The classical composition of ionic liquid-based gel electrolytes for lithium battery applications consists of polymers such as poly(vinylidene fluoride) (PVdF), polyethylene oxide (PEO), lithium salts, and ionic liquids. In a classical study, Singh et al., 2017 [70] have demonstrated a flexible gel electrolyte from PEO, lithium bis(trifluoromethane)sulfonimide (LiTFSI), and 1-Ethyl−3-methylimidazolium bis(trifluoromethylsulfonyl)imide ([EMI^+^][TFSI^−^]) with highest value of room temperature (RT) conductivity of 0.21 mS cm^−1^ at 12.5 wt.% of ionic liquids. Similarly, Ardebili et al., 2016 [71] have obtained flexible solid-like electrolytes based on poly(vinylidene fluoride-co-hexafluoropropene) (PVdF-HFP), LiClO_4_, and 1-Ethyl–3-methylimidazolium dicyanamide ([EMI^+^][DCA^−^]). The electrolytes with polymer to ionic liquid ratio of 1:2 exhibit a relatively high RT conductivity of 0.6 mS cm^−1^ combined with higher stability to organic solvent-based electrolytes which allows the simple fabrication of flexible prototype battery with demonstrated cycling at both flat and bent configurations. Several efforts were made on the synthesis of new ionic liquids and the composition of ionic liquids-based GPEs, in which pyrrolidinium-based ionic liquids have shown their great potential [72,73]. A combination of high RT conductivity (0.69 mS cm^−1^) and good flexibility under repeated bending was obtained for the mixture of 70/30/60 weight ratio of polymer host/lithium salt/ionic liquid, respectively [72]. Recently, Swiderska-Mocek et al., 2023 [73] have demonstrated that the synergy work between pyrrolidinium ionic liquids and lithium borate salts can help to stabilize the solid electrolyte interface (SEI) between gel electrolytes and lithium metal anode. The obtained GPEs are free-standing, highly flexible with high RT conductivity of 1.77–3.47 mS cm^−1^ and low SEI resistance vs. lithium metal. Ionic liquid leakages of ionogel could be prevented by the in situ crosslinking of the monomer precursors with ionic liquids and salt [41,74,75]. In another approach by doping ionic liquid in solvent-based GPEs, Montazami et al., 2015 [76] have successfully incorporated the safety of ionic liquids-based systems into those based on organic solvents without losing the performance of flexible lithium-ion polymer batteries.

Battery security requires the development of gel electrolytes with flame-resistant and self-healing properties which can be adduced by the polymer blend [77] or synthesis of new polymer structure [39,41,45]. In the first approach, cellulose acetate appears as a great candidate as an additive for both flexibility and thermal stability. Ionogel electrolytes prepared with a mixture of PVdF/PEO and cellulose acetate, which displays a conductivity of 1.06 mS cm^−1^ at 25 °C, strain at break of 198%, resisted 30 s under the flame without igniting, shrinking, or color-changing thanks to the high thermal stability of polymer and ionic liquids (N-propyl-N-methylpyrrolidinium bis((trifluoromethyl)sulfonyl)imide (Pyr13TFSI)) components [77]. Self-healable ionogel can also obtained from polymers with dynamic crosslinked bonds such as di-sulfide [39] or imine [41]. The combination of dynamic bonds and ionic liquids allows a rapid and complete self-healing effect at room temperature [41].

**Figure 1:**
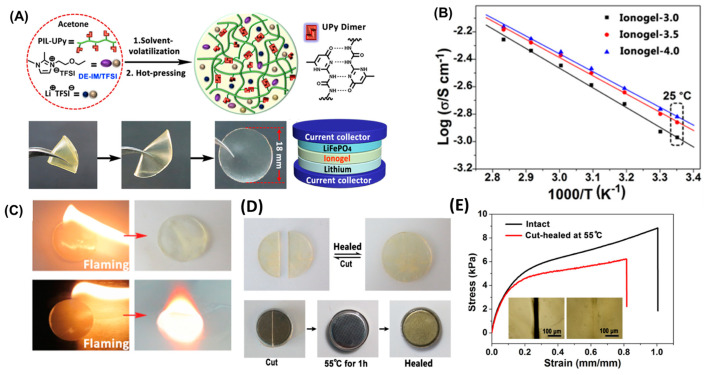
(**A**) (Top) Schematic illustration of the synthesis of PIL-UPy ionogel, (bottom) photographs demonstrating the resulting ionogel and its flexibility along with the schematic assembly of the Li-ion batteries; (**B**) evolution of the ionic conductivity as function of the temperature with different molar fractions between [DEIM^+^][TFSI^−^] and IL-UPy units (e.g., 3/3.5/4); (**C**) photographs illustrating the exposition to the fire of Ionogel−3.5 and commercial Celgard soaked with electrolyte; (**D**) photographs exhibiting the self-healing capability of the ionogel and (**E**) the stress–strain curves of as-prepared and healed ionogel. Reproduced with permission from [33]. Copyright 2019, American Chemical Society.

A mixture of poly(ionic liquids) (PILs) and ionic liquids has also gained the attention of researchers as gel electrolytes with grand potential. An improvement of GPE conductivity by replacing the neutral polymer host with poly(ionic liquid) was achieved combined with higher electrochemical and thermal stability [22]. However, a deeper understanding of the rational design of this mixture is still needed in order to optimize GPE properties [78]. Indeed, for battery applications, particularly lithium-based ones, the biggest inconvenience of ionic liquids, is their composition of both mobile cation and anion, leading to a low lithium transference number [73]. Poly(ionic liquid) can replace conventional matrix and help to immobilize part of unwanted ions. Moreover, many authors have implied poly(ionic liquids) with the same chemical nature, which induced more interaction with ionic liquids-based ions and improved lithium transference number [79,80]. In these studies, mechanically stable GPEs membrane with high conductivity (up to 1 mS cm^−1^) was achieved. Plus, the interaction between the positive charge of polyionic liquids and anions can facilitate the movement of lithium ions, thus increasing the lithium transference number in those GPEs. Poly (ionic liquids) also provide the simple addition of other functions of gel electrolytes through the modification of ionic liquid monomers. In fact, the ureido-pyrimidinone (UPy) group has been reported in the literature for its self-healing ability through intrinsic hydrogen bonding. By attaching the UPy pendant group onto polymerizable imidazolium monomer and then mixing with unmodified ILs monomer and ILs (1,2-dimethyl−3-ethoxyethyl imidazolium bis(trifluoromethanesulfonyl)imide ([DEIM^+^][TFSI^−^]), Guo et al., 2019 [33] have obtained highly conductive and flexible ionogel as presented in Figure 1. It was observed that the ionic conductivity follows Arrhenius law and evolves as a function of the ratio between the ionic liquid electrolyte ([DEIM^+^][TFSI^−^]) and the IL-UPy units. The ionogel with a ratio of 3.5 exhibits a conductivity of 1.57 mS cm^−1^ at room temperature (Figure 1B). Moreover, the ionogel displays 100% strain with extra healable and non-flammable behavior (Figure 1C–E).

A technical strategy to increase the flexibility of ionogel is to produce a 3D polymer network from electrospinning that serves as mechanical support. Electrospun-based membranes also offer more amorphous morphology and porous structure favoring the absorption of liquid electrolytes [81]. PVdF has proven its application as support for safe and flexible ionogel electrolytes [23,82,83]. Immersed in monomer and ionic liquid precursors then followed by in situ polymerization, the obtained composite-type ionogel provides remarkable performance in terms of conductivity and flexibility combined with low electrolyte leakage [23].

#### 2.1.2. Ionogels for Supercapacitor Applications

The scenario differs for flexible supercapacitors and in particular micro-supercapacitors in which high ionic conductivity and non-selective ion motion are expected. Indeed, a wide range of ionogels have been developed for this purpose. Nevertheless, the most developed ionogels for supercapacitors are based on the mixture of 1-ethyl−3-methylimidazolium [EMI^+^] based ionic liquids and common polymers such as polyvinyl alcohol (PVA) [84], PEO [85], PVdF-HFP [86]. The choice of EMI^+^ cation relies on its small size, thus relatively low viscosity compared to its analogous with long side chains. Accordingly, the yielded specific capacitance and energy density of the devices can be optimized [87]. Beyond ensuring the ionic conductivity of the gelled electrolyte, the ionic liquid can also be the plasticizer by reducing the T_g_ of the gel [88] to render free-standing ionogel. Typically, 1-ethyl−3-methylimidazolium tris(pentafluoroethyl) trifluorophosphate was mixed with PVdF-HFP (80:20 wt.%) in acetone followed by evaporation of the solvent, resulting in the free standing film of ionogel with ionic conductivity of 2 mS cm^−1^ at room temperature [89]. By assembling with two MWCNTs/PVdF-HFP/CB electrodes, the solid-state supercapacitor device exhibits an areal capacitance of 68 mF cm^−2^ (equiv. to 76 F g^−1^) as well as a specific energy of 17.2 Wh kg^−1^@18.9 kW kg^−1^. Moreover, the addition of polymeric plasticizer along with ionic liquid could extend the stretchability of the system. Adding a mixture of [BMI^+^][TFSI^−^] and Pluronic (PEO-PPO-PEO) with PEO-Li^+^ crosslinked network increases its amorphous state, yielding an ionogel (σ_i_ = 4.07 mS cm^−1^) with a stretchability reaching 29.3. When applied to µSC using MWCNTs as electrode material, an areal capacitance of 65.5 mF cm^−2^ was obtained with cycling stability over 5000 Galvanostatic charge–discharge (GCD) cycles [90].

**Figure 2:**
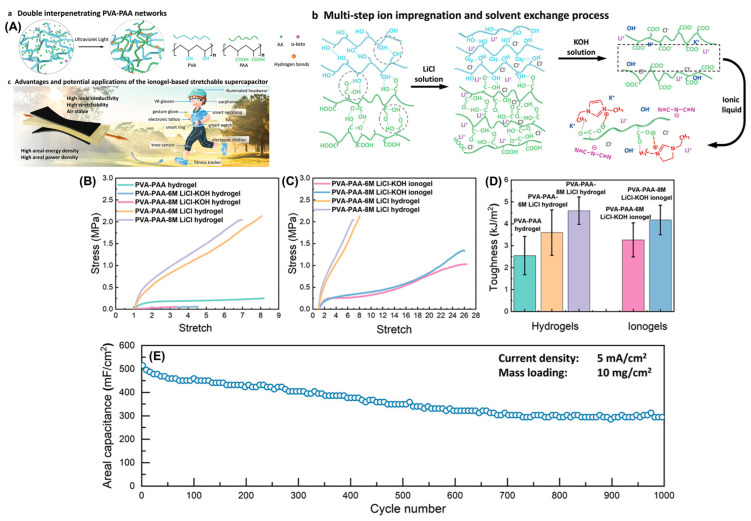
(**A**) Synthetic route of ionogel: (a) synthesis of PVA-PAA double network, (b) formation of DN hydrogel by soaking in LiCl/KOH aqueous solution and drying of the gel followed by the formation of DN ionogel by soaking in [EMI^+^][DCA^−^] ionic liquid, (c) advantages and application of ionogel based stretchable supercapacitor; (**B**) stress–stretch curves of intermediate DN hydrogels as function of electrolyte concentration; (**C**) stress–stretch curves of DN ionogels; (**D**) comparison of toughness of different involved gels; (**E**) cycling stability of the resulting SC using prepared ionogel at 5 mA cm^−2^. Reproduced with permission from [91]. Copyright 2023, Wiley.

To further increase the ionic conductivity, ionogel prepared from hydrogel was envisaged (Figure 2A) [91]. Indeed, hydrogel composed of polyvinyl alcohol–polyacrylic acid (PVA-PAA) double network (DN) containing LiCl and KOH was firstly synthetized and freeze-dried to remove water. Afterward, the freeze-dried gel was soaked in ionic liquid to yield ionogel (PVA-PAA-LiCl-KOH-([EMI^+^][DCA^−^]) with ionic conductivity reaching 0.32 S cm^−1^ at room temperature while keeping a good stretchability of 26 and a tensile strength of 1.34 MPa (Figure 2B,C). Moreover, the ionogel shows a similar toughness to the intermediate hydrogel (4.5 kJ m^2^, Figure 2D). After assembling with a porous carbon electrode, the final supercapacitor exhibits an areal capacitance of 615 mF cm^−2^ at 1 mA cm^−2^. The peak energy of 341.7 µWh cm^−2^@2 mW cm^−2^ was obtained. However, the electrochemical stability remains the weakness of the system as the capacitance retention decreases to 62% after only 1000 cycles at 5 mA cm^−2^ (Figure 2E). Most reported systems are constructed by sandwiching the ionogel membrane in between two electrodes, which may provoke an increase in the equivalent series resistance. Thus, incorporating the ionic liquid within the active electrode materials can enhance the cell performance. Zheng et al., 2019 [86] reported the pre-intercalation of ionic liquid into 2D layered materials, Ti_3_C_2_T_X_ MXene, by simple immersion of undried MXene-based µSC (thickness = 3.2 µm) into ionic liquid solution ([EMI^+^][BF_4_^−^]). Afterwards, the SC cell is removed from the ionic liquid followed by casting of [EMI^+^][BF_4_^−^]/PVdF-HFP ionogel (σ_i_ = 25 mS cm^−1^), yielding a volumetric capacitance of 133 F cm^−3^ and an energy density of 41.8 mWh cm^−3^. Moreover, the high cycling stability (>10,000 cycles at 1 mA cm^−2^) of the system was obtained without sign of performance degradation. Even though some ionogels exhibit relatively high ionic conductivities, most of them stay still at moderate value (<2 mS cm^−1^). Thus, finding new strategies to improve the ionic conductivity is still required. One possibility is to incorporate empty p-orbital elements (Lewis acid) within the gel, e.g., boron sites. Consequently, the acidic character of B-sites allows them to interact with anions, thus facilitating the ion pair dissociation. Jin et al., 2018 [92] reported the use of a network of borate ester-based dimethacrylate monomer (BEM). Indeed, the addition of BEM (12 wt.%) increases the ionic conductivity of the ionogel about 2 times at 25 °C, i.e., 5.13 mS cm^−1^ vs. 2.14 mS cm^−1^. Consequently, along with an increase in the specific capacitance, the ohmic drop during GCD cycles is significantly decreased from 244 mV to 89 mV.

**Figure 3:**
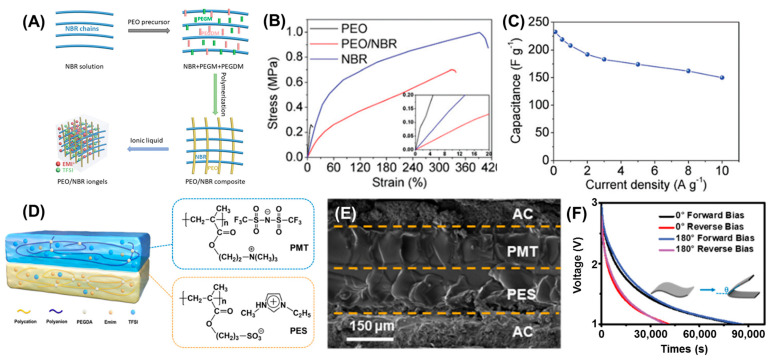
(**A**) Synthetic route to produce PEO/NBR ionogel; (**B**) stress–strain curve of PEO, NBR, and the prepared PEO/NBR ionogel; (**C**) evolution of the specific capacitance as function of the discharging current density. Reproduced with permission from [95]. Copyright 2019, Royal Society of Chemistry. (**D**) Bilayer ionogel based on PMT and PES; (**E**) SEM cross section of the SC cell; (**F**) Self-discharge profile of the bilayer ionogel with planar and folded at 180° configuration and under forward/reverse bias. Reproduced with permission from [102]. Copyright 2023, Royal Society of Chemistry.

Nevertheless, despite the simplicity of making ionogels and the increase in the electrochemical performances, in numerous cases, this strategy leads to a significant reduction of mechanical sturdiness. To compromise this issue, reticulated films appear as a better option. Nevertheless, the ionic conductivity can be strongly affected as the presence of the polymer dilutes charge concentration while reducing the ion mobility [93]. The reticulated PEO network was formed by polymerizing ethylene oxide diacrylate in the presence of 2-hydroxy−2-methylpropiophenone (HOMPP; photoinitiator) and [EMI^+^][TFSI^−^] ionic liquid (8:4:88 wt.%). The ionogel was used to fill the interspace of planar interdigitated MWCNT electrodes on a flexible Au/Ti-based PET substrate [85]. The resulting µSC has a capacitance of 5.3 F cm^−3^ at 10 mV s^−1^, equivalent to a similar µSC configuration using PVA/H_3_PO_4_ hydrogel [94]. However, with extended electrochemical operation voltages, an energy density of 2.9 mWh cm^−3^@ 50 mW cm^−3^ was obtained. Also, the stability of ionogel-based µSC is highly superior in open-air conditions with only a slight performance degradation after 2 months (~10%). Semi interpenetrated polymer network (Semi-IPN) of natural butadiene rubber (NBR) and PEO can also be envisaged as a host matrix to be swelled with [EMI^+^][TFSI^−^] ionic liquid (60 wt.%) as presented in Figure 3A [95]. The resulting ionogel has an ionic conductivity of 2.4 mS cm^−1^ and a tensile modulus of 0.69 MPa at an elongation at a break of 338% (Figure 3B). By assembling between two graphene electrodes, the generated SC displays a volumetric capacitance of ~230 F g^−1^@0.1 A g^−1^ that decreases to 150 F g^−1^@10 A g^−1^, i.e., a capacitance retention of 65% (Figure 3C). In another strategy, neutral polymers are replaced by poly(ionic liquids) to provide ionogels having mechanical robustness, high thermal stability, good flexibility, and conductivity [96]. It is noteworthy that the physical–chemical properties of poly(ionic liquids) are strongly dependent on the nature of their cations and anions. Increasing the free side chain of the cationic units decreases the ionic conductivity of the PILs (up to four orders of magnitude) [97] contrary to the effect induced by the alkyl spacer between the polymer backbone and the ionic liquid moieties [98]. Moreover, Ye et al., 2011 [99] reported the thermal stability of imidazolium-based poly(ionic liquids) with different mobile anions, and a difference of 102 K was obtained by changing the anion from Br^−^ (T_degradation_ = 534 K) to TFSI^−^ (T_degradation_ = 636 K) with intermediate values obtained from PF_6_^−^ (579 K), BF_4_^−^ (586 K) and Triflate (Tf^−^) (608 K). Concerning the cation centers, the thermal stability can be classified as follows: imidazolium~pyridinium > pyrrolidinium > ammonium [98]. A network of polyacrylamide and Poly([VMIm^+^][TFSI^−^]) was synthetized in the presence of N,N′-methylenebisacrylamide as a crosslinker and [EMI^+^][TFSI^−^] via radical polymerization. The ionogel was used as a solid electrolyte to cover PEDOT/carbon cloth electrodes. With 10% poly(ionic liquid), the ionogel can be reversibly stretched to 500% with an elongation break at 1300%. The observed ionic conductivity is ~10 mS cm^−1^ at room temperature. However, the value decays over time and remains at only 70% of its initial value after 30 days. Concerning the electrochemical performance, the assembled supercapacitor exhibits a cell capacitance of 52.3 F g^−1^@0.1 A g^−1^ and an energy density of 14.2 Wh kg^−1^@70 W kg^−1^ [100]. When crosslinked co-polymer poly([VMIm^+^][TFSI^−^])/PAAM is formed in presence of [EMI^+^][TFSI^−^] and a deep eutectic solvent, ethylene glycol/choline chloride, the ionogel with the equimolar ratio between ionic liquid and choline chloride exhibits good ionic conductivity of 5 mS cm^−1^ at room temperature with an elongation at break of 253%. Concerning the storage performance, an electrochemical window of 2.8 V and a device-specific capacitance of 43.8 F g^−1^@0.25 A g^−1^ can be obtained. Therefore, the energy density was enhanced to 35.1 Wh kg^−1^ with a capacitance retention of 94.3% after 10000 GCD cycles [101].

Besides high storage performances (capacitance, energy@power density), the self-discharge process is also among the main challenge of supercapacitors. However, almost all the reported works declined to investigate this parameter. Recently, a new strategy has been proposed by Dong et al., 2023 [102] using a bilayer of opposite-charged poly(ionic liquid) based ionogel to tackle the rapid self-discharge problem. Poly[2-(methacryloyloxy)ethyl]trimethylammonium bis(trifluoromethanesulfonyl) imide (PMT) and poly[1-ethyl−3-methylimidazolium (3-sulfopropyl)methacrylate] (PES) networks were synthetized by radical polymerization from their respective monomers in presence of PEGDA crosslinker (Figure 3D,E). The cationic PIL ionogel was placed by the positive electrode while the anionic PIL ionogel was in contact with the negative electrode. This configuration allows for obtaining an ionic barrier for preventing the reverse displacement of mobile ions, thus significantly lowering the self-discharge rate (23.2 h, Figure 3F). Moreover, this ability is maintained intact after 3000 bending cycles (at 180°).

The ionic liquid monomers can also be included in the ionogel networks as crosslinkers. Polymerization of 2-hydroxyethylmethacrylate in the presence of 1,4-di(vinylimidazolium)butane bisbromide ([DVIM^+^][Br^−^]) leads to the formation of freestanding ionogel. With 10 wt.% of [DVIM^+^][Br^−^] and 75 wt.% of [BMIM^+^][PF_6_^−^] ionic liquid, the ionogel exhibits an ionic conductivity of 0.3 mS cm^−1^ at room temperature which is much lower compared to other types of ionogels. Coupling with CNT-based electrodes, the capacitance of 3.81 mF cm^−2^ at 1 mA cm^−2^ was obtained [103].

The chemically crosslinked ionogels exhibit major limits concerning low stretchability and irreversible physical damage due to excessive deformation. To tackle these problems, physically crosslinked systems could be envisaged. Shi et al., 2020 [90] presented the use of a physically crosslinked ionogel made from double network systems, i.e., PEO-Li^+^ (1.2 M of Li^+^) network and PEO-PPO-PEO micellar network, in the presence of [BMIM^+^][TFSI^−^] ionic liquid (80 wt.%). The free-standing ionogel yields an ionic conductivity of 4.07 mS cm^−1^ at room temperature and reaches 9.8 mS cm^−1^ at 70 °C. Importantly, the safe operation voltage can reach 4 V with a strain of 2100% (tensile strength of 0.33 MPa). Due to reversible bonding between Li^+^ and polymer networks, the self-healing efficiency of 96.3% was obtained after 2 min at RT. The ionogel was then used as a substrate onto which interdigitated electrodes were directly deposited by ink writing. The areal capacitance of 65.5 mF cm^−2^@0.5 mA cm^−2^ (equiv. 21 F g^−1^) was obtained with strong cycling stability after 5000 GCD cycles. Moreover, the µSC performance remains intact after repeated cutting/self-healing cycles (5 cycles). In between rigid chemical bonding and physical bonding, dynamic covalent bonding can also be used to generate flexible and self-healable ionogel. Accordingly, boronate ester functions were employed by adding boric acid to the PVA solution [84]. The resulting gel, with an elongation at break of 176% and an ionic conductivity of 2.43 mS cm^−1^ (90 wt.% of [EMI^+^][Cl^−^]), possesses high healing efficiency of 96.89% after 21 cycles of cutting/healing. By using AC-based electrodes, the assembled SC yields an energy density of 10 Wh kg^−1^@0.9 kW kg^−1^ and can be self-healed at 95% after 30 min.

### 2.2. Hydrogel

Hydrogels refer to soft and water-insoluble three-dimensional networks of polymer that can nevertheless absorb a large amount of water (up to 99 wt.%) [104]. As with other types of polymer networks, two categories of hydrogels can be identified, including chemically crosslinked and physically crosslinked networks. Indeed, the first category consists of hydrogels formed by chemical reactions via radical polymerization for example [105,106]. The second category of hydrogels can be originally generated by means of various types of interactions (ionic, hydrophobic, molecular recognition, self-assembly, etc.) [107]. Thanks to a large amount of water or aqueous electrolyte contained in the gel, the ionic conductivity of hydrogel represents the highest values among different families of gel electrolytes reaching the S cm^−1^ range [108].

#### 2.2.1. Hydrogels for Supercapacitor Applications

Compared to the battery applications, the hydrogels are widely used in supercapacitors. Since the last decade, around 1500 articles have been published, representing around 50% of reported works on the use of gel electrolytes for supercapacitor application. Indeed, with numerous developed hydrogels for supercapacitors, it would be more convenient to classify them by their main provided functionalities, including (1) flexibility/stretchability/compressibility, (2) antifreeze/antidrying, and (3) self-healing characteristics. It is noteworthy that these three functionalities do not compromise among themselves. Flexible by nature, and stretchable by material engineering, while many hydrogels possess these characteristics, their integration into performant flexible and stretchable supercapacitors faces multiple challenges. The first issue relates to the aqueous electrolyte that interferes with the storage process by side reactions (water splitting or oxygen reduction reaction, etc.). Thus, the operation voltage is usually restrained, which limits the stored energy. The second challenge consists of the non-equity of mechanical properties between the pristine hydrogel and the electrodes as well as the adhesion of the gel with the electrode. Last but not least, the electrochemical storage performance seems to form a dilemma with the stretchability and self-healing functions. Thus, multifunctional supercapacitors owning all the characteristics have not yet been reported and remain the future of the field. Nevertheless, within this review, state-of-the-art systems will be summarized to provide a clear landscape of the hydrogel for energy storage applications.

Concerning deformable (flexible, stretchable, compressible) supercapacitors, the most common systems consist of the combination of linear polymer chains of PVA, polyacrylamide (PAM), PAA, etc., and acid/base/salt crosslinking agents. The popularity of these hydrogels relies on the versatility of the synthesis in which the polymers are mixed in aqueous acid/base/salt solutions. Accordingly, when the research focus is on the development of active electrode materials with integration in flexible supercapacitors, this type of hydrogel is systematically used. Despite the provided flexibility, they suffer from major problems, including rapid water evaporation, and relatively low ionic conductivity due to low water retention and restraint mechanical properties. To partially tackle this problem, dual crosslinked systems and double network (DN) hydrogels have been developed.

**Figure 4:**
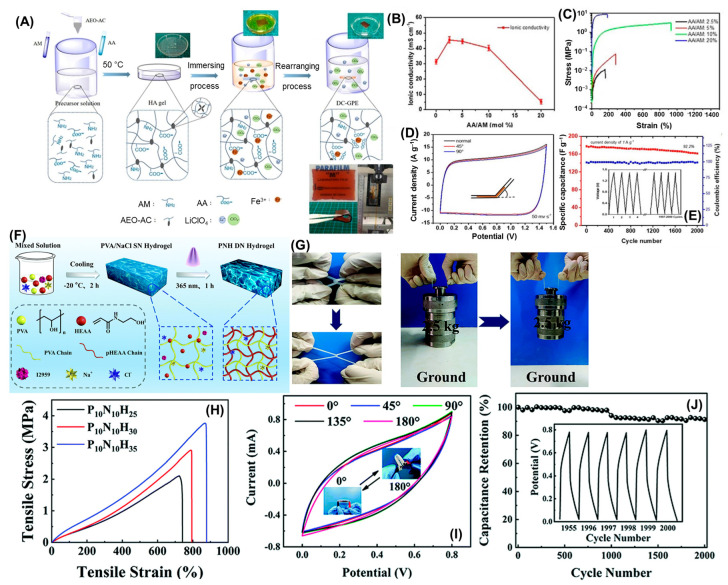
(**A**) Synthetic pathway to prepare PAM/PAA dual crosslinked network; (**B**) evolution of the ionic conductivity and (**C**) stress–strain curves of the GPE as function of the ratio of the two monomers; (**D**) voltammograms of the flexible SC at different bending angles; (**E**) cycling stability of the SC. Reproduced with permission from [111]. Copyright 2020, Wiley. (**F**) Scheme illustrating the formation of PVA/PHEAA double network hydrogel; (**G**) photographs presenting the flexibility and stretchability of the gel; (**H**) stress–strain curves of DN gels with different wt.% of HEAA monomer (25, 30, 35 wt.%); (**I**) voltammograms of the optimized DN hydrogels at different bending angle up to 180° and (**J**) cycling stability of the gel. Reproduced with permission from [113]. Copyright 2022, Royal Society of Chemistry.

In addition to the primary function of being flexible and stretchable, some systems provide self-healing ability originating from dynamic crosslinked structures. The ionic conductivity can reach 0.35 S cm^−1^ by using a PAA/PAM chemically crosslinked network in the presence of modified lignin as a source of dynamic physical interactions (hydrogen bonding and coordination bonding) [109]. Using activated carbon as electrodes, the resulting supercapacitor exhibits a specific capacitance of each electrode of 210 F g^−1^@1 A g^−1^. The high rate capability with 87.2% in capacitance retention at 5 A g^−1^ is comparable to a liquid-based system. As mentioned, the increase in the electrochemical performance reduces therefore the stretchability of the film, reaching only ~700%. By scarifying the ionic conductivity and energy storage performances, hydrogel made from poly(AMPS-co-DMAAm) crosslinked with both laponite and graphene oxide [110] exhibits a stretchability of 1000% and a conductivity ranges from 6 mS cm^−1^ to 30 mS cm^−1^ depending on the composition. Assembled in between two CNTs@polyaniline electrodes, the resulting supercapacitor exhibits an areal capacitance of 180 mF cm^−2^ (9 mF cm^−2^ when only CNTs are used as electrodes). Moreover, the capacitance (normalized by the surface area of the device) remains intact with an elongation at 900%. By replacing the chemical crosslinking molecule with a physical crosslinker, alcohol ethoxylate, the PAA/PAM network can be obtained followed by the second crosslinked system using complexation between carboxylate functions and Fe^3+^ as presented in Figure 4A [111]. The resulting dual physical network exhibits an elongation at break at 1000%, a tensile strength of 3.1 MPa, and an ionic conductivity of 40.1 mS cm^−1^ (Figure 4B,C). A specific capacitance of 178.5 F g^−1^@0.5 A g^−1^ was obtained using activated carbon as active electrode material. The capacitance remained intact with different bending angles (reported up to 90°, Figure 4D). Moreover, the SC exhibits a capacitance retention of about 92% after 2000 GCD cycles (Figure 4E). Full chemically dual crosslinked hydrogels, even though less reported, have also been investigated. Guo et al., 2020 [112] reported a network of PVA/PEO using glutaraldehyde as a crosslinker via acetalization reaction. Without owning reversible physical bonding, the gel does not possess high stretchability and self-healing ability. Nevertheless, good electrochemical performances were obtained with ionic conductivity of 67.1 mS cm^−1^ and specific capacitance of 773 mF cm^−2^@0.2 mA cm^−2^ (polypyrrole/polyaniline electrodes). Beyond the cycling stability in a planar configuration, the capacitance retention remained at 100% after 1000 bending cycles (180°).

The second strategy, double network hydrogels start to attract attention as efficient gel electrolytes in supercapacitor applications. As commonly known, DN hydrogels are special interpenetrating polymer networks (IPNs) that differ from conventional IPNs as they own two contrasting polymer networks within their structure and the second polymer could have a molar concentration of 20–30 times higher than the primary one [114,115], resulting in nonlinear correlation in properties between the DN hydrogel and their respective single networks. Indeed, a typical DN hydrogel consists of a first minor component of a rigid skeleton made from highly crosslinked networks and a second and major portion of a ductile component (poorly crosslinked network). Accordingly, the resulting DN hydrogels simultaneously possess high mechanical strength with high elongation at break (>500%) and strong toughness with high tensile strength (>1 MPa) [114,116]. Different crosslinked DN hydrogels can be envisaged. A fully physically crosslinked DN hydrogel based on PVA and poly(N-hydroxyethyl acrylamide) (PHEAA) was developed in which both networks are made from physical interactions with NaCl or by hydrogen bonding (Figure 4F) [113]. Nevertheless, with the salt-out effect, the PVA network appears as a rigid one while the PHEAA remains ductile. The optimized conditions lead to a DN hydrogel with ionic conductivity of 0.13 S cm^−1^ with a tensile strength of 3.76 MPa and a good stretchability with an elongation at break of 872% (Figure 4G,H). The supercapacitor assembled using the resulting gel and two MWCNTs/ITO/PET electrodes shows an areal capacitance of 3 mF cm^−2^ at 0.05 mA cm^−2^. The performance was kept intact upon bending up to 180° (Figure 4I). The cycling stability of 90% after 2000 GCD cycles was observed (Figure 4J). Lin et al., 2018 [117] reported the use of a physically–chemically crosslinked DN hydrogel, Li-AG/PAM, as an electrolyte for supercapacitors. Indeed, within the voids of agarose contained Li_2_SO_4_ single network, the acrylamide monomer, and N,N′-methylene-bis-acrylamide crosslinked agent were filled and polymerized under γ-ray irradiation (initiator free system). Depending on the Li^+^ and SO_4_^2−^ concentrations, the toughness and the stretchability of the film can be modulated with tensile strength maximum reaching 1.4 MPa and the best elongation at break reaching 3608% ([Li_2_SO_4_] = 0.3 mol kg^−1^). The mechanical properties downgrade at higher concentrations. With the same concentration of inorganic salt in the hydrogel, the ionic conductivity is about 20 mS cm^−1^ with a maximized value at 45.7 mS cm^−1^ ([Li_2_SO_4_] = 1.25 mol kg^−1^). As a dilemma between mechanical properties and ionic conductivity is established, the best-compromised system (σ_T_ = 1.1 MPa, λ_T_ = 2780%, σ_i_ = 41 mS cm^−1^) was found with 0.94 mol kg^−1^. The constructed SC using activated carbon coated nickel foam as electrodes yields a specific capacitance of 84.7 F g^−1^ (single electrode) along with an energy density of 1.7 Wh kg^−1^@50 W kg^−1^. The device also shows good capacitance retention upon deformations, including compression (90% capacitance retention after 1000 compression cycles at 50% strain) and bending (100% capacitance retention after 1000 bending cycles at 90°). Coordination chemistry can also be employed to yield a dynamic network. Within the chemically crosslinked PAM network, alginate chains were physically crosslinked via complexing carboxylate functions with Al^3+^ ions. The hydrogel shows moderate toughness with a tensile modulus of 430 kPa with a high energy dissipation of 137.8 kJ m^−3^. Consequently, the hydrogel and later, the assembled supercapacitor device exhibit superior tolerance to mechanical constraints (twisting, compressing, squeezing, folding, hammering) while retaining the capacitance intact (94.7 mF cm^−2^@0.1 mA cm^−2^). Recently, redox-active molecules have been incorporated into DN hydrogels to enhance the electrochemical storage performance. Typically, Na_2_MoO_4_ was added in a solid-state gelatin PHEAA chitosan DN hydrogel (tensile stress of 1.08 MPa and elongation at break of 1200%) [118]. To access the electroactivity of the MoO_4_^2−^ and to increase the ionic conductivity of the gel, different acids were also incorporated. Thus, by using activated carbon-based electrodes, the areal capacitance of 84 mF cm^−2^@1 mA cm^−2^ (420 F g^−1^) was obtained along with an energy density of 34 Wh kg^−1^@1.8 kW kg^−1^. The system displays good flexibility as a function of bending angle up to 180°.

**Figure 5:**
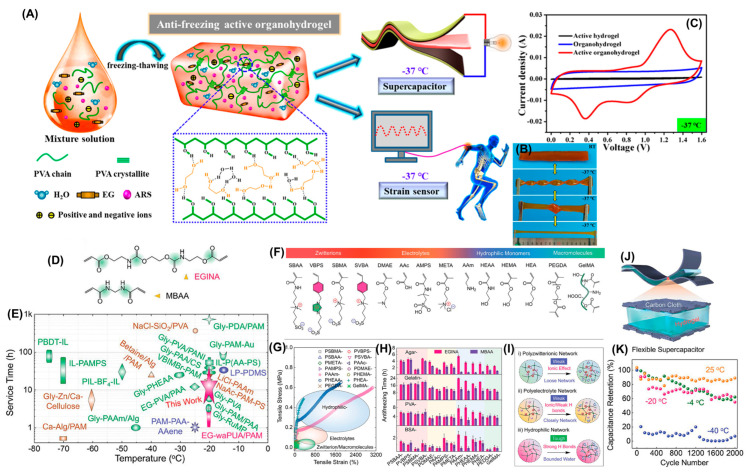
(**A**) Scheme illustrating the formation of PVA organo-hydrogel using EG/H_2_O binary solvent and its potential applications in supercapacitor and strain sensor; (**B**) photographs showing the flexibility and stretchability of the organo-hydrogel at −37 °C; (**C**) voltammograms of the assembled SC at −37 °C using different gel electrolyte. Reproduced with permission from [128]. Copyright 2021, American Chemical Society. (**D**) Chemical structure of MBAA (N,N′-methylenebisacrylamide) and EGINA (4,9-dioxo−5,8-dioxa−3,10-diazadodecane−1,12-diyl diacrylate); (**E**) positioning of the reported anti-freezing hydrogel using EGINA crosslinker compared to other reported systems in terms of service time and temperature; (**F**) different monomer structures used to synthetize anti-freezing hydrogels; (**G**) stress–strain relation and (**H**) anti-freezing time of hydrogel resulted from monomers reported in (**F**); (**I**) possible interactions within the GPE; (**J**) scheme of the SC devices and (**K**) capacitance retention over 2000 GCD cycles at different temperatures. Reproduced with permission from [133]. Copyright 2021, Wiley.

It is noteworthy that as the water molecules, the major component of hydrogels, are captured within a polymeric network, they exist as free water or bound water molecules depending on their interactions with the polymeric chains [119]. With a large amount of free water molecules, the majority of hydrogels lose their mechanical properties and cannot operate at temperatures below 0 °C due to the formation of ice. Nevertheless, incorporating anti-freezing agents in the formulation of the gel could unblock the issue and pave the way for soft-wet hydrogel in cold conditions. The final goal of this agent is to provide high interactions with water molecules to disturb/disrupt the water lattices at sub-zero degrees. Depending on the mechanism of preventing ice nucleation, anti-freezing hydrogels can be commonly classified into two main categories, ionic antifreezing hydrogels, and antifreezing organo-hydrogels. Besides, nanocarbon can also be incorporated into the hydrogel to ensure antifreezing function [120,121]. Taking advantage of the colligative effect of ionic compounds, the ionic hydrogels incorporate inorganic salts with high concentrations to lower the freezing point of the gel. Thus, generating hydrogel rich in hydrophilic functions with high ionic adsorption capacity allows to facilitate salt dissociation, thus increasing the interaction with free water molecules [122,123,124]. However, the presence of salting-in-salts like CaCl_2_ certainly increases the antifreezing ability but at the same time compromises the mechanical properties of the hydrogel [125]. For supercapacitor applications, recent works have started to shed light on the elaboration of antifreezing hydrogel electrolytes. Zhang et al.,2023 reported the use of a zwitterionic network, composed of [2-(methacryloyloxy)ethyl]dimethyl-(3-sulfo-propyl)ammonium hydroxide (SBMA) and N-(2-Hydroxyethyl) acrylamide. The hydrogel was soaked in 6 M of LiCl until reaching equilibrium. By incorporating the LiCl, the melting peak of water strongly shifts from −2.6 °C to −47.3 °C followed by a decrease in the melting enthalpy more than 3 times (from −70 to −19 J g^−1^), confirming the crucial role of dissociated salt in the gel. The yielded hydrogel shows moderate stretchability with elongation at a break of 500% and a high ionic conductivity at 2.58 S cm^−1^ at room temperature. More interestingly, the ionic conductivity remained at a reasonable value of 0.22 S cm^−1^ at −20 °C. Between the two temperatures, the specific capacitance, measured with MXene/graphene oxide electrodes, was reduced by 29.3% from 218.2 F g^−1^@1 A g^−1^ to 154.2 F g^−1^ with good reversibility after 6 freezing—thaw cycles (at −20 °C and −40 °C). To further increase the number of hydrogen bonding and the dissociation of LiCl, poly(amidoxime) fragments were grafted to PSBMA chains, and allyl glycidyl ether was used as a crosslinker [126]. An ionic conductivity of 3 S cm^−1^, 1 S cm^−1^ and 0.34 S cm^−1^ were obtained at 30 °C, −20 °C and −30 °C. Accordingly, Ti_3_C_2_T_x_ MXene-based SCs exhibit high specific capacitance of 225 F g^−1^@1 A g^−1^ (520 F cm^−3^) with good capacitance retention above 90% after different deformations (compressing at 80% strain, twisting, and bending at 90°). Even though a natural decrease in the capacitance was observed, i.e., 317 F cm^−3^ at −30 °C), the capacitance retention after 1000 stretching cycles (at −30 °C and 60% strain) remained high, at 80% of the initial value. In a second approach, binary solvents (organic/water) are used to lower the freezing temperature. It should be noted that the presence of organic solvent reduces the solubility of inorganic salts in the hydrogel. Among different choices, glycerol and ethylene glycol (EG) are widely used. Moreover, the presence of these molecules induces noncovalent interactions with polymeric networks, leading to an increase in the toughness of the gel by participating in energy dissipation. Rong et al., 2018 [127] reported the mixture of water and ethylene glycol as a dispersion medium in the PVA network. Using 33.3 wt.% of EG, the mechanical properties of the hydrogel can be kept intact at −40 °C with an elongation at a break of 300% and a tensile strength of 125 kPa. However, the ionic conductivity drops from 16 mS cm^−1^ to ~3 mS cm^−1^. Accordingly, the areal capacitance decreases by 30% as the temperature drops from room temperature to −40 °C. Nevertheless, the SC device exhibits good flexibility at different bending angles (up to 180°) and good cycling stability even at −20 °C with a capacitance retention of 88% after 5000 cycles. To improve the storage performance, redox molecules (alizarin red S) were added to the PVA/H_2_SO_4_/H_2_O/EG organo-hydrogel (Figure 5A,B) [128], leading to a specific capacitance of 350 F g^−1^@0.5 A g^−1^ at room temperature and 240 F g^−1^ at −37 °C (Figure 5C). The capacitance retention at −37 °C is about 91% after 5000 GCD cycles and 97% after 50 days, indicating no degradation in the performance was observed. Besides the use of ethylene glycol, other organic solvents can also be used to render organo-hydrogel with antifreezing properties, such as dimethyl sulfoxide (DMSO) [129,130], glycerol [131,132], etc. Recently, antifreezing hydrogels without any additives have been developed by using up-to-date 4,9-dioxo−5,8-dioxa−3,10-diazadodecane−1,12-diyl diacrylate (EGINA) as a crosslinked agent [133]. Zhang et al., 2021 [133] reported the use of EGINA crosslinker to generate different DN hydrogels, ranging from zwitterionic monomers to neutral macromolecules as presented in Figure 5D–H. Depending on the network, the mechanical properties can be drastically changed with elongation at break ranging from 70% to 1200% at −20 °C (Figure 5G). As a typical example, HCl-doped PVA/PHEAA@EGINA exhibits an elongation at a break of about 500% and a conductivity at −20 °C of 0.1 S cm^−1^ (0.2 S cm^−1^ at room temperature) with antifreezing service time around 6 h (Figure 5H). An areal capacitance of 45 mF cm^−2^@10 mA cm^−2^ was obtained at −20 °C with a capacitance retention of 74.8% compared to the value at 25 °C (Figure 5K). Because of its structure, EGINA is highly hydrophilic and short enough to provide tight networks that force the free water molecules to interact with the polymer matrix, thus intrinsically reducing the freezing point of water (Figure 5I) [134].

Besides reported hydrogels with antifreezing properties, thermoresponsive hydrogels have started to emerge as a new approach for the elaboration of smart SC devices. Two types of systems can be envisaged, including heat-protective and heat-activated devices. For the first system, Tan et al., 2023 [135] reported the use of poly(N-isopropylacrylamide) (polyNIPAM) hydrogel with 4,4′-(dodecane−1,12-diyl)bis-(1-vinyl−4H-imidazolium)bromide as crosslinker. Indeed, the polyNIPAM is known as a low critical solution temperature thermoresponsive polymer with a phase transition temperature of about 25–60 °C depending on the molar weight, the morphology, and the presence of additional charges [136]. Accordingly, above the gel-gel transition temperature of 60 °C, compression of the gel was observed, and the specific capacitance of 100 F g^−1^ dropped to zero, indicating a blockage of the ion conduction channels and the capture of ions within the polyNIPAM structure within the hydrogel. Accordingly, the diffusion coefficient of Li^+^ ion decreases by two orders of magnitude when the temperature changes from ambient (5.97 × 10^−7^ cm^2^ s^−1^) to 60 °C (2.72 × 10^−9^ cm^2^ s^−1^). Interestingly, the change in the electrochemical responses is highly reversible with 100% retention after several heating/cooling cycles. On another side, the compression of the polyNIPAM can be profitable to generate electrical percolation when active electrode material is incorporated within the gel. Elashnikov et al., 2021 [137] proposed to mix polypyrrole (PPy) during the formation of the hydrogel, leading to randomly unconnected PPy nanotubes inside the gel. With 5 wt.% of PPy, the thickness of the device changes about three times with stimulation (heat/light), leading to an increase in the conductivity of one order of magnitude. Accordingly, the capacitance after stimuli (40 °C or under illumination at 780 nm) increases from 30 F g^−1^ to 360 F g^−1^. The commutation times are around 50 s (thermal activation) and 30 s (light activation).

#### 2.2.2. Hydrogels for Battery Applications

Hydrogel is not commonly used in battery applications since water can affect the performance of the battery (parasitic reaction with electrode materials and evaporation of water) [138]. One strategy reported in the literature is to fabricate water in salt electrolytes in which the solvation process of salt at high concentrations helps to avoid water evaporation, thus increasing the battery’s long-term stability. The same approach was conducted by Huang et al., 2019 [139] utilizing sodium polyacrylate as a polymer matrix for NiCo/Zn battery. Lower water content and higher ion concentration permit high conductivity (0.6 mS cm^−1^ at −20 °C, 1.3 mS cm^−1^ at 24 °C) and remarkable mechanical strength and strain of obtained hydrogel combined with antifreezing and antiheating effects. Stable cyclability under long cycling and upon different bending over a large range of temperatures from −20 to 50 °C was also observed [139].

**Figure 6:**
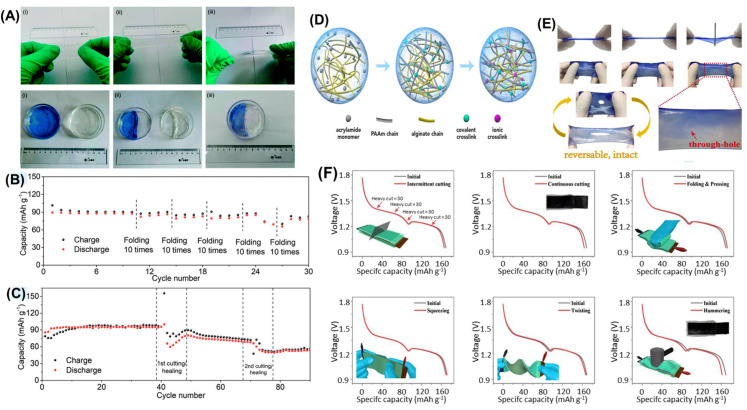
(**A**) The PZM hydrogel: (top) (i,ii) before–after stretching and (iii) twisting; (bottom) (i) two pieces of PZM gel (one colored in blue for visuality), (ii) two-half parts and (iii) self-healed PZM gel; (**B**) evolution of the capacity as function of cycle number and folding cycles; and (**C**) evolution of the capacity as function of the cycle number and healing cycles. Reproduced with permission from [31]. Copyright 2022, Royal Society of Chemistry. (**D**) Formation of the PAAm hydrogel; (**E**) photographs displaying the mechanical properties of the hydrogel (flexible, stretchable); (**F**) the polarization curves of the assembled Zn-MnO_2_ battery under mechanical constraints. Reproduced with permission from [144]. Copyright 2019, Elsevier.

Another challenge of hydrogel in flexible batteries is their low mechanical strength. The choice of polymer matrix becomes then crucial. Among commonly used polymer matrices, PAM and polyacrylate offer high compressibility and flexibility [140,141]. Sodium polyacrylate gel electrolytes permit the fabrication of an intrinsically 400% stretchable and 50% compressible NiCo//Zn battery with enhanced or well-retained electrochemical performance [140]. As previously discussed, PVA is often used to create hydrogel electrolytes for supercapacitors with high flexibility issued from hydrogen bonding between water and PVA. Recently, Liu et al., 2022 [31] have demonstrated an interest in using PVA hydrogel combined with Zn^2+^ and Mn^2+^ ions allowing self-healing on flexible electrolytes for zinc-ion batteries (Figure 6A). Coupling with flexible electrodes, the battery presents high stability upon repeated bending, folding, twisting, and cutting/healing at 90 °C (Figure 6B,C). Development of new smart polymers such as poly(N-isopropylacrylamide) has recently gained attention since this thermal-gated polymer structure exhibits hydrophilic-hydrophobic transition of gel electrolytes at high temperatures allowing the halt of ionic migration through the gel electrolytes, avoiding cell thermal runaway and protecting battery at high temperature [54]. Natural polymers have also gained attention in flexible batteries such as the use of gelatin [138], or xanthan gum [142]. Liu et al., 2018 [138] have demonstrated the interesting application of gelatin as a polymer matric for durable and flexible self-standing electrolytes for zinc metal batteries. Gelatin-based hydrogel’s considerable porosity allows for the reserve of high electrolyte content leading to an ionic conductivity as high as 6.15 mS cm^−1^ at room temperature. The high mechanical strength of gelatin-based hydrogel prevents Zn dendrite formation. Thus, a steady Zn plating/stripping performance was observed with gelatin-based electrolytes combined with a high and stable capacity of 110 mA h g^−1^ for solid-state Zn/LiMn_2_O_4_ battery. Zinc-ion battery using xanthan gum mixed ZnCl_2_ as gel electrolytes features high 201 mA h g^−1^ at −20 °C, with cycling stability over 1500 cycles and 92% capacity retention over 100 cycles at bending state [142]. Mechanical properties can also be improved by crosslinking the polymer matrix. Thanks to the synergy of covalent and reversible bonding (e.g., hydrogen bonding), hydrogels exhibit elastomeric-like behavior with higher water absorption crosslinked, thus higher ionic conductivity. A compressible Zn-MnO_2_ battery was successfully obtained using crosslinked PAM hydrogels with a performance of maximum specific capacity of 230.5 mA h g^−1^ at 1C and stability over 1000 cycles at 4C and unchanged charge–discharge performance upon compression [143]. The use of bio-based polymer with possible ionic crosslinks as alginate sodium has also gained interest since covalent and ionic crosslinks systems have shown their potential in improving mechanical performance of hydrogel. An impressive hydrogel obtained using a Zn-alginate/PAM crosslinked polymer matrix (PZM) (Figure 6D,E) exhibits high modulus and fracture toughness, which helps to prevent dendrite formation in Zn-based batteries [144,145]. A Zn/MnO_2_ battery using this aforementioned hydrogel as gel electrolytes presents unchanged electrochemical performance under different shape and size deformation (Figure 6F) [144]. Using similar gel electrolytes in sodium-ion/Zn battery, high cycling stability with a low capacity loss of 0.0027% per cycle over 10,000 cycles was reported by Huang et al., 2020 [145].

Even though promising results have been recently reported, the hydrogels have faced the major problem of being dehydrated while working extensively under dried conditions. Moreover, the dehydration/rehydration cycles are completely absent to the best of our knowledge. Thus, a real demonstration of operational hydrogel-based devices in arid environments has still not yet been reported.

### 2.3. Organogels

As indicated by their name, organogels are composed of polymeric networks filled with organic solvents. Different types of networks have been reported for the elaboration of organogels [146], including low-molecular organogelators (LMOGs), polymeric organic gelators (POGs), and polymer networks with strong affinity to the organic solvent.

#### 2.3.1. Organogels for Battery Applications

**Figure 7:**
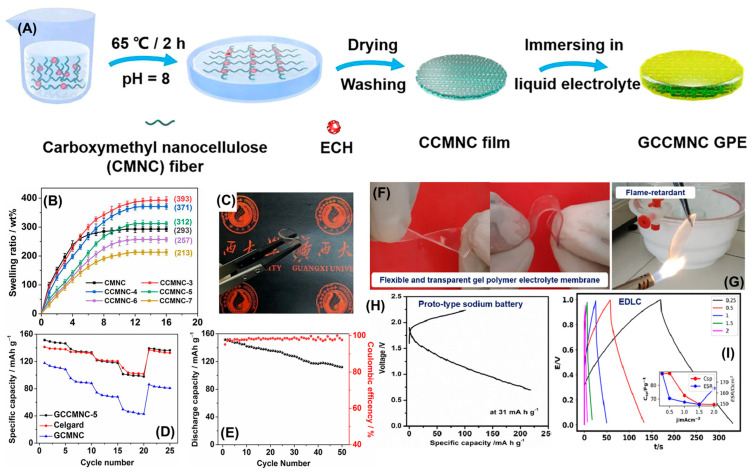
(**A**) Schematic procedure for synthetizing GCCMNC GPE; (**B**) electrolyte uptake and swelling of the gel with different thicknesses; (**C**) photograph of the flexible film; (**D**) evolution of the discharge capacity as function of the C-rate (0.2, 0.5, 1, 2C) and of the cycle number; (**E**) cycling stability of the Li/GCMNC−5 GPE/NCM523 cell at 0.2C. Reproduced with permission from [150]. Copyright 2022, Elsevier. (**F**) Photographs of PVdF-HFP/NaPF_6_/TMP organogel showing the flexibility of the GPE; (**G**) flame-retardant characteristic of the GPE; (**H**) polarization curve of Li/GPE/LFP cell at 31 mA g^−1^; and (**I**) galvanostatic charge–discharge curves of the SC with inset figure the evolution of the specific capacitance as function of the current density. Reproduced with permission from [152]. Copyright 2022, Elsevier.

Widely reported in the literature, organogels are dominant as GPE for battery applications. For lithium-based batteries, organogels are preferable due to the water sensibility of electrodes. In a conventional system, polymer matrices are often swollen in liquid electrolytes containing carbonate solvents, e.g., ethylene carbonate, propylene carbonate, and dimethyl carbonate, and lithium salt such as LiPF_6_ at 1 M. To develop a simple way to prepare GPE electrolytes under air, Scrosati et al., 2015 [147] have obtained a self-standing and easy-to-handle GPE based on PVdF and EC:DMC: LiPF_6_ electrolytes with high conductivity of 0.5 mS cm^−1^ at 30 °C and low interfacial resistance versus graphite and LiFePO_4_ electrodes. Like other types of gel electrolytes, the choice of polymer matrix and the crosslink are crucial to increase flexibility and stretchability. By using a semi-interpenetrating network based on poly(methyl acrylate-co-acrylonitrile)/poly(vinyl alcohol), the obtained GPE containing 150% of EC:DMC:LiPF_6_ electrolytes displays a conductivity of 0.98 mS cm^−1^ at 30 °C combining with high fracture strength of 14.3 MPa and high elongation at break of 250% [148]. Others focus on the use of rubber-like crosslinked polymer (cross-linked polyepichlorohydrin network) which allows highly stretchable gel electrolytes (300% elongation) with acceptable conductivity at room temperature (0.24 mS cm^−1^) [149]. Among bio-based polymers, crosslinked cellulose has shown its potential in organogel featuring high conductivity (4.4 mS cm^−1^), excellent mechanical strength (67 MPa), and high flexibility. An exceptional lithium transference number of 0.90 was observed owing to the presence of polar functional groups of cellulose-based organogel [150,151]. To achieve high Li-transference numbers, Wang et al., 2022 [150] used carboxymethylated nanocellulose as a host polymer (GCCMNC) from which a crosslinking process was performed in the presence of epichlorohydrin (ECH), leading to flexible GPE (Figure 7A,C). With low concentrations of ECH, the prepared gel can be swelled in the electrolyte up to 393%, and thus an ionic conductivity of 3.93 mS cm^−2^ (Figure 7B). Thus, the resulting gel was used in Li-ion batteries, showing a discharge capacity of 151.4 mA h g^−1^@0.2C (Figure 7D) which is superior to other cellulose-based electrolytes. However, a high-capacity fade of about 0.52%/cycle (Figure 7E) was observed.

UV-induced in situ polymerization [153,154,155,156] has been recently favored as a simpler way to prepare gel electrolytes with the advantages of reducing gelation time, eliminating solvents, and reducing side reactions [156]. With only 30 s under UV, Gao et al., 2022 [156] synthesized an ethoxylated trimethylolpropane triacrylate network containing LiTFSI solvated with tetraglyme with high conductivity (0.63 mS cm^−1^) and robust mechanical strength. A similar ionogel was synthesized by Bhattacharyya et al., 2022 [155] from poly(ethylene glycol) methyl ether methacrylate with enhanced ionic conductivity (2.3 mS cm^−1^) and mechanical stability.

Similar to the adopted approach for ionogel, the self-healing capability of organogel electrolytes can be provided through quadruple hydrogen bonding of UPy pendant groups [34,35]. UPy-grafted monomers were synthesized and copolymerized with PEG-based monomer resulting in crosslinked organogel for lithium-ion battery with good flexibility, high mechanical strength, high ionic conductivity (0.75 mS cm^−1^, RT), and self-preparation without external stimulation [34]. Moreover, high interfacial stability versus Lithium metal was also observed with stable voltage polarization of about 0.01 V over 400 h in lithium symmetric cells. Concerning fire resistance behavior, the choice of thermally stable polymer (e.g., blend of PVdF and poly(1,2-diethoxyethylimidazolium TFSI (PDEIm) [157] or smart thermally responsive polymer (poly(acrylonitrile-co-diethyl vinylphosphonate) [P(ANDEVP)] [158] and high flash point solvent such as fluoroethylene carbonate (FEC) [46,47] are crucial. Phosphorous-based flame retardant additives can also be incorporated as supplemental plasticizers (Figure 7F,G) [152] or covalent grafted to polymer networks [159] aiming to enhance the fire resistance of gel electrolytes. Flexible, non-flammable, and highly conductive gel electrolytes were obtained leading to excellent long-cycle stability of full lithium-ion cell (graphite as anode and LiFePO_4_ as the cathode) with an average capacity attenuation rate of only 0.057%, which functions correctly after being bent, winded, folded, punched and clipped.

#### 2.3.2. Organogels for Supercapacitor Applications

Unlike hydrogels, pure organogels are scantily reported as gel electrolytes for supercapacitor applications, especially flexible SC, issuing from the toxicity, inflammability, and recyclability of organic solvents that require specific encapsulation of the devices. In a more convenient way, organohydrogels are usually used as reported in the previous section. Nevertheless, few studies have reported the elaboration of organogel for supercapacitor applications. Besides using as battery GPE, PVdF-HFP/NaPF_6_/TMP gel can also be used for thermoresponsive supercapacitor applications [152]. A specific capacitance of 100 F g^−1^ at 10 mV s^−1^ was obtained when activated carbon was used as electrodes (Figure 7I). The stability of the GPE was also demonstrated with 4000 GCD cycles. Zheng et al., 2022 [160] reported the organogel based on using PVdF-HFP as a polymer matrix blended in acetonitrile, methyl formate, and propylene carbonate. The combination renders multiple hydrogen bonds, including H-F, H-N, and H-O hydrogen bonds, which lead to a free-standing membrane. However, [EMI^+^][BF4^−^] ionic liquid was used as salt. As the gel is composed of component owning low freezing point, the electrochemical performance at sub-zero temperature is still enough for SC application. The ionic conductivity of 9.21 mS cm^−1^ and 2.95 mS cm^−1^ at ambient temperature and −60 °C were obtained which are comparable to other categories of anti-freezing gels. Moreover, the mechanical properties of the organogel are perfectly maintained for a wide range of temperatures, ranging from room temperature to −40 °C with a stretchability of 400% and a tensile strength at 2.2 MPa. The interest of using organogel relies on large operation voltage which is extended to 3 V for the reported system. Using activated carbon-based electrodes, symmetric SC was assembled with a specific capacitance of 120 F g^−1^@0.5 A g^−1^ (single electrode capacitance). However, the rate capability of the device decreases with the temperature drop. Chemically crosslinked can also be envisaged. PVdF-HFP/PVP was soaked in trimethylolpropane triacrylate followed by crosslinking by γ-ray irradiation [161]. The resulting GPE exhibits good flexibility with an ionic conductivity of 0.06 mS cm^−1^ (14.4 mS cm^−1^ after being soaked in acetonitrile containing tetraethylammonium tetrafluoroborate) at ambient. Moreover, the membrane is displayed as a good flame-retardant material. Once assembled, the fabricated SC exhibits a safe operation voltage of 2.9 V and a specific capacitance of 41.3 F g^−1^@1 mA cm^−2^ (mass of two electrodes). Recently, PVA-based organogel with ionic conductivity of 45.45 mS cm^−1^ was obtained by incorporating trimesic acid and phenylenediamine in DMSO [162]. Besides the hydrogen bonding between different functions, the presence of a Lewis acid–base couple renders acid–base reactions to yield ionic interactions. Thus, the mechanical properties can be enhanced with an elongation at a break of ~450% (tensile strength of 2.5 MPa). For storage performance, a specific capacitance of 322.5 F g^−1^@0.1 A g^−1^ was obtained with only a slight change in the performance upon bending (up to 180 °C). However, the faradaic current was obtained while activated carbon was used as active electrode material. It could originate from the redox activity of small molecules in the gel, e.g., phenylene diamine. Thus, even though high capacitance was obtained, the cycling stability of this system may be the subject of further investigations.

### 2.4. Single-Ion Gel Electrolytes

The design of single-ion conducting electrolytes is crucial for battery application as high cation transference numbers can prevent cell polarization and dendrite nucleation and growth [163,164,165]. Simple single-ion conducting gel electrolytes can be obtained for zinc-ion batteries due to the double charge of zinc ions, which allows the physical cross-link of the natural polymer network and the formation of gel electrolytes. From a similar approach to the aforementioned hydrogels, Chan et al., 2021 [164] have synthesized a DN hydrogel based on iota carrageenan and polyacrylamide. As a result of Zn^2+^ ionic crosslinks on fixed sulfates anion onto iota carrageenan polymer chain, a high Zn^2+^ transference number of 0.93 was obtained for this gel electrolytes combining with an excellent ionic conductivity of 2.15 mS cm^−1^ at room temperature and faire mechanical properties (tensile strength of 123 kPa and stretchability over 300% strain) [164].

For lithium base batteries, the main principle of preparing single-ion conducting gel electrolytes is to prepare the single-ion conducting polymer with attached anion and lithium counteraction and subsequently boost their conductivity by adding organic solvent as a plasticizer. The synthesis of the single-ion conducting polymer was conducted either by ionic covalent grafting was effectuated in different polymers (PVdF [165], polyimide [166]) or copolymer (poly(ethylene-co-vinyl alcohol) [167]) or the formation of polymer network using polymerizable ionic contained monomer (lithium 2-acrylamido−2-methylpropane sulfonate [168] or lithium 4-Styrenesulfonimide [169]). In all these studies, except for the network obtained by Xu et al., 2020 [169] where supplement lithium salt was added leading to a lower transference number of 0.6, all gel electrolytes possess a transference number close to unity, an acceptable conductivity of around 0.1 mS cm^−1^ at room temperature and good mechanical behavior. For example, single-ion conducting gel electrolytes based on polyimide exhibits a tensile strength of 30 MPa and Young’s modulus of 1.72 GPa [166].

### 2.5. Hybrid Gel Electrolytes

#### 2.5.1. Hybrid GPEs for Battery Applications

The compromise between ionic conductivity and mechanical properties is crucial for flexible gel electrolytes for battery applications. One of the strategies is to mechanically reinforce the polymer matrix using inert fillers such as silica (SiO_2_ [170,171,172,173], polyoctahedral silsesquioxanes POSS [174]), TiO_2_ [175], or Al_2_O_3_ [176] in searching for higher conductivity through higher electrolytes uptake without endangering their mechanical performance. A recent study on several types of inert nanofillers has revealed better interfacial stability provided by Al_2_O_3_ nanofillers [176] but no improvement in flexibility was observed. Many studies favor the addition of silica nanofillers owing to their large surface area and good interfacial properties versus organic matrix [172]. In fact, SiO_2_-reinforced gel electrolytes provide higher conductivity than unmodified ones independently from the polymer nature (PVdF-HFP [170,173], poly(vinyl chloride) (PVC)/poly(ethyl methacrylate) (PEMA) blend [172] or PIL [177]) because of higher electrolytes uptakes and lower polymer crystallization. A conductivity of close to 1 mS cm^−1^ at 25 °C could be obtained combined with high tensile strength (6.72 MPa) and elongation (80%) [170]. Flexible lithium-ion battery also presents high specific capacity and high tolerance to mechanical abuse [173].

**Figure 8:**
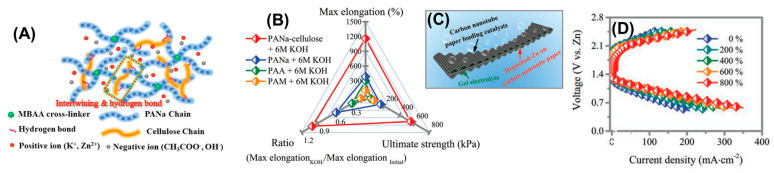
(**A**) Schematic representation of PANa/cellulose hydrogel; (**B**) radar chart representing the mechanical characteristics of the hydrogels in function of their compositions; (**C**) Schematic representation of the flexible Zn-air battery; (**D**) polarization curves at different elongations of Zn-air battery using PANa/cellulose/6 M KOH hydrogel as GPE. Reproduced with permission from [22]. Copyright 2019, Wiley.

Cellulose nanofibrils (CNFs) have been naturally used as efficient agents for improving the mechanical behavior of hydrogel electrolytes as a result of their high Young’s modulus and tensile strength. Moreover, the hydrophilic nature and the chemical structure with abundant hydroxyl groups of cellulose help to improve the ionic conductivity once incorporated in hydrogel electrolytes [22,177,178,179]. Typically, a tough hydrogel can also be obtained by blending cellulose (crosslinked with 1,4-Butanediol diglycidyl ether) and bentonite (10 wt.% of cellulose), resulting in strong interactions between them. A compressive strength of 3.2 MPa and fracture energy of 0.45 MJ m^−3^ were obtained. By doping with LiCl, the final hydrogel exhibits an ionic conductivity of 89.9 mS cm^−1^, which remains at 25.8 mS cm^−1^ at −20 °C [122]. CNFs were largely incorporated in hydrogel electrolytes (Figure 8A) for lithium-ion [179], zinc-ion [178], zinc-air [22], and aluminum-air [177] batteries in which they provide excellent conductivity (>100 mS cm^−1^) and high elongation (up to 1200%, Figure 8B). The presence of CNFs also provides the stretchability of gel electrolytes with excellent stability of battery-specific capacity after 100 cycles of stretch/release tests at 800% (Figure 8C,D) [22]. Recently, cellulose was also added into ionogel electrolytes as a flexible host framework structure [180,181].

Unconventional fillers such as nanoclays and covalent organic frameworks were recently employed in the development of new hybrid gel electrolytes. In fact, nanoclays such as lithium montmorillonite have been widely used as mechanical reinforcing agents for polymer matrices since exfoliated layers of clays can intercalate polymer chains and reduce crystallinity. Together with its ability to transfer lithium ions, the incorporation of lithium montmorillonite can upgrade the conductivity of gel electrolytes for lithium batteries [182] (up to 0.48 mS cm^−1^ at 25 °C). Covalent organic frameworks have recently gained their attention as promising materials for energy storage applications. Incorporating covalent organic framework fillers in ionogel electrolytes improves their ionic conductivity owing to good interaction of ILs and covalent organic frameworks which allow higher IL uptake, better dissociation of ILs, and faster diffusion of ions through covalent organic framework structures [183].

In recent years, many researchers have delved into the utilization of conducting fillers such as graphene oxide (GO) [184,185] and conducting ceramics [186,187,188,189] in searching the synergy of ionic conductivity and mechanical performance. The presence of GO in polymer matric improves interaction with liquid electrolytes (1.0 m LiPF_6_ in EC/DEC = 1:1) and increases electrolytes uptake and retention, which as a result increases the conductivity of the final gel electrolyte. Higher dissociation of lithium-ion and immobilization of counter anion due to hydrogen bonding could also improve lithium-ion transport [185]. Coupling with LiCoO_2_ as cathode and Li_4_Ti_5_O_12_ as anode, the full lithium-ion cell presents high electrochemical stability (better capacity at 140.4 mAh g^−1^) after 100 thousand bending release cycles [184]. In recent years, polymer/ceramic hybrid electrolytes using conducting ceramics such as Li_0.35_La_0.55_TiO_3_, Li_1.3_Al_0.3_Ti_1.7_(PO_4_)_3_, or Li_6.4_La_3_Zr_1.4_Ta_0.6_O_12_ have gained increasing as a good combination of high conductivity of ceramic and flexibility of polymers for lithium battery application. Percolation of ceramic materials at high concentrations of ceramics materials is necessary to ensure ionic conduction paths. Three-dimensional frameworks appear to be a good solution to obtain up to 60 wt.% of ceramics in gel electrolytes in preserving their flexibility but low conductivity at room temperature of these gels was attained (lower than 0.1 mS cm^−1^) [188,189]. In fact, a recent study has revealed the optimized concentration of Li_6.4_La_3_Zr_1.4_Ta_0.6_O_12_ of 25 wt.% in poly(vinylidene fluoride-tri-fluoroethylenechloro fluoroethylene) to maximize gel electrolytes conductivity. An excellent RT conductivity of 1.8 mS.cm^−1^ was obtained due to the reduction of the crystallization zone on the polymer matrix and continuous conduction pathways all provided by the incorporation of active ceramic materials. Interestingly, the authors have obtained an ultra-flexible lithium battery that loses only 7% of its initial capacity after suffering 150,000 times mechanical bending [187].

Nanofillers dispersion is crucial to optimize the improvement of desired properties. One strategy to tackle the dispersion issue is a chemical modification of inorganic fillers with organic chains to increase compatibility [190,191]. Indeed, SiO_2_-g-p(methyl methacrylate-co-hydroxyl ethyl methacrylate) nanoparticles synthesized by grafting copolymer onto the surface of SiO_2_ provide better dispersion of nanofillers in PVdF matrix leading to higher electrolytes (1 M LiClO_4_ in PC) uptake. As a result, a high ionic conductivity of 2.63 mS cm^−1^ at 298 K was obtained [190]. Chen et al., 2022 [174] have succeeded in crosslinking POSS nanofiller into the polymer matrix to create new hybrid ionogel electrolytes with remarkable conductivity (2.5 mS cm^−1^ at 25 °C) and desired mechanical properties (2.6 MPa for stress, 90% for strain). Other research focuses on the in-situ crosslinking of the polymer matrix in the presence of nanofiller and liquid electrolyte [178,191]. Zhi et al., 2018 [178] have successfully performed in situ polymerization of polyacrylamide to form nanofibrillated cellulose-reinforced hydrogel for Zn-MnO_2_ hydrogel. The resulting electrolytes are highly conductive at room temperature (22.8 mS cm^−1^), super flexible (1100% strain), and sewable. A smart design of single-ion conducting hybrid gel electrolytes containing multi-ionic conduction channels was obtained by Jia et al., 2022 [192] from self-assembly and in situ polymerization of aerogel and polyethylene glycol diacrylate (PEGDA). Possessing good mechanical properties (stretchable up to 78% and compressible over 50% without any fracture), high ionic conductivity of 0.25 mScm^−1^ at room temperature, and a high transference number of 0.96, this electrolyte demonstrates a stable cycling in lithium symmetric cell (240 h at 0.4 mA cm^−2^).

The addition of nanofillers is also advantageous for other required properties of flexible electrolytes such as self-healing [40] or flame retardancy [48,193,194,195,196]. A three-dimensional cross-linked network prepared by Xie et al., 2024 [40] from different methacrylate monomer and thiolene click reactions. POSS nanoparticles not only mechanically reinforce the gel electrolytes but also serve as crosslinked nods allowing the insertion of self-healing monomer containing di-sulfide dynamic bond. The obtained gel electrolytes present acceptable conductivity (7.71 × 10^−4^ S cm^−1^ at 30 °C), excellent interface stability (no short circuit in Li symmetric cell for more than 800 h), and a stable cycling capacity of 126 mAhg^−1^ up to 960 cycles at 0.5C in Li metal cell using LiFePO_4_ as a cathode. The incorporation of inorganic fillers type SiO_2_ [193,195] or TiO_2_ [48] improves the thermal stability of gel electrolytes, thus enhancing their flame resistance. SiO_2_ nanoparticles have been proven to protect the polymer matrix during combustion owing to the ability to migrate to the polymer surface and to form a dense and uniform silicon-containing carbon protective layer [195]. Hence, Zou et al., 2018 [193] have benefited from the hydrogen bond between polyurethane and aerogel SiO_2_ in preparing multifunctional gel electrolytes (high conductivity, flexibility, and flame resistance). Using this electrolyte, the Li–O_2_/air full batteries exhibit stable charge–discharge behavior upon continuous bending and twisting conditions or under oxygen. On the other hand, the use of inorganic flame-retardant additives such as black phosphorus nanosheets (BPn) is advantageous in improving not only the security of lithium-ion batteries but also ionic conduction and mechanical tensile performance (strain at break of over 200%) [197]. In situ cross-linking of the polymer matrix (PMMA) in the presence of BPn helped to achieve a more homogenous distribution of ILs, lithium salt, and BPn nanofillers allowing high conductivity (1.08 mS·cm^−1^ at 30 °C), flexibility upon repeated folding and high electrochemical stable (550 h cycling at 0.2 mAcm^−2^ in lithium symmetric cell without an interior short circuit) [197].

#### 2.5.2. Hybrid GPEs for Supercapacitor Applications

Concerning supercapacitor applications, few works have been devoted to the development of hybrid gel electrolytes. A dual crosslinked PAA polyelectrolyte composed of two crosslinking mechanisms was reported [198], including chemical bonding with vinyl-terminated silica nanoparticles and physical interactions via hydrogen bonding. After soaking in phosphoric acid and with a water uptake at 60 wt.%, the ionic conductivity can reach ~8 mS cm^−1^. Moreover, the hydrogel with only 0.1 wt.% of Si nanoparticles presents a stretchability of more than 3700%. The gel with 83.5 wt.% in water was placed in between two electrodes made from polypyrrole-coated carbon nanotube paper to obtain a supercapacitor device with a stretchability of 600%, probably limited by the mechanical limit of the electrodes. In an unstretched state, the specific capacitance of a single electrode was obtained at ~210 F g^−1^ at 5 mV s^−1^ while the value increases about 2 times at 600% elongation. Moreover, the dynamic hydrogen bonding within the gel allows also the ability for self-healing properties. Even though the stretchability is significantly reduced with healing cycles (stretchability of 76% after the 6th cycle), the electrochemical performances seem not to be affected with capacitance retention of around 100% after 20 self-healing cycles. In another approach, Zheng et al., 2022 [129] reported the use of graphene as an inorganic filler in a PVA organohydrogel (DMSO/water/H_2_SO_4_). The tensile strength of the gel is highly enhanced compared to conventional PVA-based gels, reaching 2.58 MPa and an elongation at break at 300% while possessing an ionic conductivity of 0.39 S cm^−1^ at ambient (0.1 S cm^−1^@−65 °C). The graphene/PVA gel was sandwiched between two PANI/carbon cloth electrodes to afford the flexible SC with a specific capacitance of 253 F g^−1^ with superior capacitance retention of 93% at −65 °C. Being photoactive, TiO_2_ nanoparticles were introduced in the gel precursors mixture ([BMI^+^][BF_4_^−^], DMAAm, and MBAA) to serve as photo-initiator with their holes from the exciton formation under UV irradiation and to ensure the mechanical reinforcement [199]. The resulting ionogel has an ionic conductivity of 3.5 mS cm^−1^ at room temperature (20.1 mS cm^−1^ at 200 °C). Assembled with activated carbon electrodes, a specific capacitance of 143 F g^−1^@1A g^−1^ was obtained. The value not only increases with increasing temperature (177 F g^−1^ at 200 °C) but also with compression (~160 F g^−1^ at 3.2 MPa). Moreover, the specific capacitance remains intact at different bending angles to 90°. Wu et al., 2021 [200] reported an organogel made from PVA mixed in shear thickening fluid (STF) along with using SiO_2_ filler as GPE for wide operation temperature flexible supercapacitors. Depending on the STF percentage, the ionic conductivity of the gel could be tuned from 1.6 mS cm^−1^ (60% STF) to 2.8 mS cm^−1^ (0% STF) and can reach 7 mS cm^−1^ at 80 °C. It should be noted that all the compositions display good stretchability over 300%. Thus, a cell capacitance of 21 F g^−1^@1 A g^−1^ was obtained at ambient and the value doubles at 80 °C. Moreover, the organogel can dissipate impacts up to 570 N with 88% capacitance retention.

In addition to the development of mechanically reinforced gels as electrolytes and separators of SC devices, different studies reported the incorporation of electrode materials within the formulation of the gel, resulting in all-in-one gel electrodes inheriting the mechanical properties of the gel systems. Hu et al., 2017 [201] reported the elaboration of Poly(vinyl alcohol)/PANI hydrogel as an electrode for a flexible SC device. Accordingly, the hydrogel was prepared in a two-step process in which the porous PVA gel was first prepared followed by incorporation and polymerization of aniline within the porosity of the first network. The resulting hydrogel exhibits good mechanical properties with an elongation at a break of 696% (tensile strength of 0.51 MPa). The specific capacitance of the electrode of 150.5 F g^−1^@2 mV s^−1^ was obtained (equiv. to 300.9 mF cm^−2^ or 4.3 F cm^−3^). The capacitance retention remained above 90% after 100 bending cycles at 180 ° and also after 10,000 GCD cycles in an unbent state. More recently, Peng et al., 2023 [202] proposed another formulation based on the PVA/PANI system. Indeed, poly(vinyl alcohol) was mixed with aniline, ammonium sulfate (oxidant), and super P additive in an acidic aqueous solution to form gelled electrodes. By using a PVA–sulfuric acid electrolyte in between two PANI/PVA/SP electrodes, a specific capacitance of 550 F g^−1^@0.2 A g^−1^ was obtained with good cycling stability. The SC device provides a self-healing ability and can operate under bending by maintaining the storage performance. More complex systems could also be conceived to enhance the electrochemical storage performances of the rendered SC devices. A gelled electrode made from complexly crosslinked Ti_3_C_2_T_X_ MXene-coated PANI nanofiller, gelatin, PAAm (crosslinkers = Fe^3+^, phytic acid, methylene-bis-acrylamide) was reported by Han et al., 2024 [203] The resulting electrode (thickness = 0.5 mm) owns an elongation at break at 1300% (tensile strength of 28.8 kPa). An areal capacitance of 827 mF cm^−2^@1mA cm^−2^ was obtained with contribution from both redox active components, i.e., MXene/PANI and Fe^3+^. Considering the mechanical properties of the gel, the specific capacitance was found intact at different bending angles up to 180°. More importantly, the reported formulation allows the SC to operate both at sub-zero temperature with capacitance retention of 55% at −30 °C and high temperature with 150% capacitance retention at 85 °C. Instead of homogeneously blending electrode materials with the gel electrolyte, unidirectional macroporous AgNWs/Ag-CNTs aerogel was used as the host matrix to be filled with organohydrogel (PAM/EG/H_2_O) [204]. The electrode exhibits as a consequence high conductivity of 965 S cm^−1^, a stretchability of 2430% at ambient. More interestingly, the mechanical properties are only slightly affected by the temperature with an elongation at break decreases to 2100% at −35 °C (1080% for the full SC cell) and to 1970% at 80 °C (1010% for the full SC cell), indicating a wide operational temperature window. The areal capacitance of 168 mF cm^−2^@0.5 mA cm^−2^ was obtained at −35 °C along with good capacitance retention (85% at 9 mA cm^−2^). Moreover, the device exhibits a strong cycling stability with a capacitance retention of 97.1% after 10000 GCD at −35 °C which remains as stable after cutting/healing cycles (128 mF cm^−2^ after 20 cycles).

## 3. Conclusions and Future Prospects

To tackle the problems linked to the liquid electrolyte towards applications in flexible and wearable electronics, the development of GPE is becoming more than ever crucial. In the literature, three main families of GPE can be easily identified based on the solvent captured inside the polymer matrices, namely hydrogel, ionogel, and organogel. Each of them has specific characteristics that allow them to be complementary to each other. As mentioned throughout the review as well as the summarized table (Table 1), many attempts have been made to render new GPEs with improved performances (better thermal stability, ionic conductivity, flexibility/stretchability, electrochemical window, ion transference number, etc.). More recently, self-healing and thermoresponsive materials have started to emerge in the field of GPE as a new functionality to the existing systems. Therefore, a combination of different GPEs has been envisaged, such as prepared ionogel from pre-existing hydrogel or utilizing binary/ternary solvents, leading to an enlargement of the GPE possibility. Moreover, to enhance the electrochemical storage performances, redox moieties (e.g., Alizarin Red-S, MoO_4_^2−^) have also been incorporated inside the GPE, leading to a contribution of pseudocapacitance to the supercapacitor applications. However, the main problem of the GPE relies always on low ionic conductivity and the dilemma between the ionic conductivity and other functionalities. Thus, with the rapid advancement in wearable electronics, it is highly required to develop more efficient GPE for energy storage systems.

Even though GPEs have widely been attractive in energy-related fields, the commercialization of these membranes is still far in view of the recent state of the art. Three major technical problems could be identified: the long-term stability of the GPEs, their encapsulation, and their fabrication costs. Firstly, even though different terms, including stable, highly stable, and superior stability have been used in case no sign of degradation is detected during the testing process in lab-scale devices and under controlled environments. Nevertheless, industrial-oriented tests have not yet been applied concerning all of the reported GPEs, except for commercially available Celgard membranes. Accordingly, the expected lifespan of storage devices should range from 2 to 7 years (equivalence to 500–2000 cycles for batteries) [205]. Common failure mechanisms could be (i) the drying and volatilization of the solvent within the GPEs, i.e., water and volatile organic solvents; (ii) the uneven potential changes in two electrodes during charge/discharge cycles; (iii) uneven mechanical stresses at two sides of the cell; and (iv) the side reactions (e.g., degradation of electrolytes). The second challenge consists of the fabrication cost of the well-designed GPEs. In 2022, the average price of a battery is about USD 150 per kWh, including ca. 10–15% of separator and electrolytes as reported by the Internal Energy Agency. Nissan Motor Corporation has forecasted a drop in the price of solid-state batteries to USD 75 per kWh by 2028 and USD 65 per kWh in the next years. However, the production cost of GPEs is much higher than this benchmark value. As an example, for ionogels, EMITFSI’s price is about USD 700 kg^−1^ (industrial grade) and poly(ionic liquids) are still not commercially available. In addition to other costs (utilities, maintenance, labor, etc.), the prices of chemicals and materials occupy about 70% of the total cost and some of the common chemicals are presented in Table 2. Thus, reducing the price of the GPEs is equivalent to the replacement of expensive chemicals as [EMI^+^][TFSI^−^] or MBAA with more cost-effective ones.

The third challenge relies on the encapsulation of the GPE-based devices. As the reported GPEs exhibit high performance with outstanding mechanical properties, encapsulating them with encapsulation materials will reduce their performance as the encapsulation materials must have higher mechanical performances than the GPEs to not be the limiting factor. However, common materials used for this purpose are silicone, polyurethane, epoxy-based resin, etc., which do not have as high mechanical properties as the GPEs inside.

For future directions, incorporating nanofiller to render hybrid GPE to enhance the mechanical properties of the gel while maintaining the electrochemical performances can be envisaged. It should be noted that even though the fruitful development of incorporating nanofillers in GPEs during the last two decades, the understanding of interfacial interactions between the complex GPE matrix and nanofiller materials is not fully established to render a general guideline to achieve high-performance flexible energy storage devices, especially in light of industrial application. Consequently, future research should focus on the efficient combination of experimental performance with theoretical calculations which are still challenging due to the complexity of the GPE composition and interactions. Nevertheless, unlocking this bottleneck would lead to the identification of the optimal route to elaborate GPE composites owing to maximized interfacial interaction between the polymer chains and the surface of the filler while preventing or minimizing the agglomeration of the components. Moreover, it is noteworthy that even though GPEs exhibit a highly promising future, most of them are combined with electrodes with inferior mechanical performances, leading to trivial overall mechanical responses of the storage devices. Accordingly, an all-in-one approach using a unique GPE throughout the cell can be considered as a good option to tackle this problem while always keeping in mind the problem of electrical conductivity of the active electrode materials once dispersed inside the polymer matrices. However, the interface between electrically conductive materials and ionically conductive materials is another challenge as a poorly defined interface would lead to a long and tortuous ion diffusion pathway. Thus, the interfacial resistances can be undesirably increased, and the performances of the active materials could be significantly impacted. For most of the reported works, conventional materials (e.g., NMC and activated carbon for batteries and supercapacitors, respectively), have been used as default choices without considering this aspect. Therefore, the compatibility between the GPE and the electrode material needs to be more deeply investigated. Last but not least, even though GPE can be considered as solid or quasi-solid electrolytes, exogenous electrolytes are required in almost all systems to ensure good ionic conductivity of the final gel. Thus, developing a new concept of intrinsically ionic conductive GPE would be promising to yield a real solid electrolyte system based on GPE.

## Figures and Tables

**Table 1:** Recently reported gel electrolytes for flexible energy storage systems with their application and properties.

Applications	Gel Electrolytes Composition			Gel Properties					Energy Storage Devices		Reference
	Gel Polymer Network	Electrolyte	Crosslinker and Others	Mechanical Properties				Ionic Conductivity (mS cm^−1^) at RT	Electrode Material	Capacitance /Capacity	
				Flexible	Stretchable	Self-Healable	Thermo Responsive				
**Ionogel**											
**BATTERIES**	PEGMA/PVdF-co-HFP *electrospun*	1 M LiTFSI [EMI^+^][BF_4_^−^]	PTA	√	x	x	x	1.7	LiFePO_4_ Li metal	160 mA h g^−1^ @0.1 C	[23]
	PIL-UPy copolymer	[DEIM^+^][TFSI^−^]/ LiTFSI		√	√	√	x	1.57	LiFePO_4_ Li metal	147.5 mA h g^−1^ @0.2 C	[33]
	PEG-IP−2SS	[EMI^+^][TFSI^−^]	4-arm PEG HPDS/IPDI	√	√	√	x	1.75	3DMC	42.6 mF cm^−2^ @0.4 mA cm^−2^	[39]
	PEO	LiTFSI (20 wt.%) [EMI^+^][TFSI^−^] (12.5 wt.%)		√	x	x	x	2.08	LiMnO_2_ Li metal	120 mA h g^−1^ @0.1 C	[70]
	PVdF-HFP	LiClO_4_ (10 wt.%) [EMI^+^][DCA^−^] (60 wt.%)		√	x	x	x	0.6	LiCoO_2_ Li_4_Ti_5_O_12_	300 µAh cm^−2^ @0.25 C	[71]
	PVdF-HFP	LiTFSI (30 wt.%) [PMPyrr^+^][TFSI^−^]		√	x	x	x	0.69	LiFePO_4_ Li metal	151 mA h g^−1^ @0.1 C	[72]
	PVdF-HFP	0.4 LiBOB [MePrPyr^+^][NTf_2_^−^]	TMS	√	√	x	x	1.77	LiFePO_4_ Li metal	135.15 mA h g^−1^ @0.1 C	[73]
	PVdF	1 M LiPF_6_ [EMI^+^][Tf^−^] EC/PC		√	x	x	x	2.34	LiMnO_2_ Graphite	91.8 mA h g^−1^ @125 mA cm^−2^	[76]
	PVdF-HFP PEO	LiTFSI [Pyr_13_^+^] [TFSI^−^]	CA	√	√	x	√	1.06	LiFePO_4_ Li metal	162.9 mA h g^−1^ @0.2 C	[77]
	Poly(MMA-b-MUBI-TFSI)	100 mM LiTFSI/ [EMI^+^][TFSI^−^]		√	x	x	x	10	LiCoO_2_ Li_4_Ti_5_O_12_	112 mA h g^−1^ @0.1 C	[79]
	PVdF/3P(MPBI-TFSI)	LiTFSI [EMI^+^][TFSI^−^]		√	√	x	√	~0.78	LiFePO_4_ Li metal	151 mA h g^−1^ @0.1 C	[82]
	PVdF/PMHBI-TFSI *electrospun*	0.5 M LiTFSI [EMI^+^][TFSI^−^]	PEGDA	√	√	x	x	1.3	LiFePO_4_ Li metal	152 mA h g^−1^ @0.1 C @40 °C	[83]
	P(DADMA)TFSI	LiTFSI [PYR_1,2O1_^+^][TFSI^−^]	TEOS (*precursor of SiO_2_ NPs*)	√	√	x	x	0.58	LiFePO_4_ Li metal	150 mA h g^−1^ @0.5C	[171]
	Poly ([VI-NH2^+^] [TFSI^−^])-co-PBA	[BMI^+^] [TFSI^−^]	POSS PEGDA	√	√	x	√	2.5	LiFePO_4_ Li metal	160.5 mA h g^−1^ @0.5C	[174]
	PVdF-HFP	LiTFSI/ [PYR_1,3_^+^] [FSI^−^]	Li-MMT clay	√	x	x	x	0.48	LiCoO_2_ Li metal	1 mA h cm^−2^ @0.4 mA cm^−2^	[181]
	PDADMATFSI	LiTFSI/ [PYR_14_^+^] [TFSI^−^]	TTB-DMTP-COF	√	x	x	x	0.28	LiFePO_4_ Li metal	160 mA h g^−1^ @0.5 C	[183]
**SUPERCAPACITORS**	Pluronic F127	[EMI^+^][BF_4_^−^] LiBF_4_		x	x	x	√	Not provided	AC	40 F g^−1^@ 100 °C 17.4 F g^−1^@ 14 °C	[45]
	PVA	[EMI^+^] [Cl^−^]	Boric acid	√	√	√	x	2.43	AC electrodes	90 F g^−1^ @0.1 A g^−1^	[84]
	PEGDA	[EMI^+^][TFSI^−^]	PEGDA	√	x	x	x	Not provided	MWCNT electrode (*µSC*)	5.3 F cm^−3^ @10 mV s^−1^	[85]
	PVdF-HFP	[EMI^+^][BF_4_^−^]	MXene (all-in-one) No MXene	√	x	x	x	2.2 × 10^6^ 25	MXene (all-in-one) (*µSC*)	44 F cm^−2^ @0.1 mA cm^−2^	[86]
	PVA	KI EC [EMI^+^][BF_4_^−^]		√	x	x	x	Not provided	AC	160 F g^−1^ @5 mV s^−1^	[87]
	PVdF-HFP	[EMI^+^][FAP^−^]		√	x	x	x	2	MWCNTs	90 F g^−1^ @1 mA cm^−2^	[89]
	PEO/Pluronic	1.2 M LiTFSI [EMI^+^][TFSI^−^]		√	√	√	x	4.07	MWCNTs	65.5 mF cm^−2^ @0.5 mA cm^−2^ (*µSC*)	[90]
	PVA/PAA	LiCl KOH [EMI^+^][DCA^−^]		√	√	x	x	3180	PAN derived porous carbon fibers	615 mF cm^−2^ @1 mA cm^−2^	[91]
	PEO	1 M LiClO_4_ [EMI^+^][BF_4_^−^]	BEM (15 wt.%)	√	x	√	√	5.13	rGO	34.35 F g^−1^ @1 A g^−1^ (*total mass of electrodes*)	[92]
	PEO/NBR	[EMI^+^][TFSI^−^]	PEGDM	√	√	x	x	2.4	Graphene	208 F g^−1^ @1 A g^−1^	[95]
	PAAm/PVMITFSI	[EMI^+^][TFSI^−^]	MBAA	√	√	x	x	13	PEDOT	157.8 F g^−1^ @0.4 A g^−1^	[100]
	PAAm/PVMITFSI	EG/ChCl [EMI^+^][TFSI^−^]	MBAA	√	√	x	x	~4.5@RT 13.3@90 °C	AC	32 F g^−1^@RT 43.8 F g^−1^@90 °C @0.25 A g^−1^ (*total mass of electrodes*)	[101]
	PMT/PES bilayer	[EMI^+^][TFSI^−^]	PEGDA	√	x	x	x	0.18	AC	32.1 F g^−1^ @0.5 mA g^−1^ (*total mass of electrodes*) *Self-discharge time of 23.2 h*	[102]
	PHEMA	[BMI^+^][PF_6_^−^]	DVIMBr	√	x	x	x	0.3	CNTs	25 mF cm^−2^ @0.2 mA cm^−2^	[103]
	PDMAA *On mask*	[BMI^+^] [BF4^−^]	MBAA TiO_2_ NPs	√	√	x	x	10@RT	AC	143 F g^−1^@RT 177 F g^−1^@200 °C @1 A g^−1^	[199]
**Hydrogel**											
**BATTERIES**	PANa-Cellulose	6M KOH/ H_2_O	MBAA 0.2 M Zn^2+^	√	√	x	x	280	CNTs@CC Zn metal	800 mA h g^−1^ @5 mA cm^−2^	[22]
	PVA	H_2_O	Zn^2+^/Mn^2+^	√	x	√	x	170	ZVO/CC Zn	96 mA h g^−1^ @0.1 A g^−1^	[31]
	PANa	H_2_O 6 M KOH	Fe^3+^/Zn^2+^	√	√	√	x	Not provided	NiCoO/CC Zn/CC	247 mA h g^−1^ @1 C	[32]
	CBH	20 mM KOH/ H_2_O	DVS	√	x	x	x	1.63	Zn/MnO_2_	11 mA h g^−1^ @0.5 mA g^−1^	[56]
	Pluronic/PNIPAM/AM-grafted copolymer	0.5 M H_3_PO_4_/ H_2_O		x	x	x	√	7.3 (sol) 0.07 (gel)	GO/CNT/AC	73 F g^−1^@RT 3.6 F g^−1^@80 °C (*at 10 mV s^−1^*)	[58]
	P(ICZn-AAm)	H_2_O	MBAA 0.1 M Zn^2+^	√	√	x	x	2.15	V_2_O_5_/CNT Zn metal	322.3 mA h g^−1^ @0.5 C	[159]
	NFC/PAM	H_2_O	MBAA 2M Zn^2+^ 0.2 M Mn^2+^	√	√	x	x	22.8	MnO_2_ Zn metal	200 mA h g^−1^ @4 C	[178]
	PEG/ Cellulose	H_2_O	epichlorohydrin	√	√	x	x	3.31	NMC532 Li metal	159.3 mA h g^−1^ @0.2 C	[179]
**SUPERCAPACITORS**	Pluronic/PVA	1 M KCl/ H_2_O	Borax	√	√	√	x	25.6	MWCNTs	801.9 mF cm^−3^@20 µA cm^−2^	[21]
	PVA	EG/ Glycerol H_2_SO_4_ H_2_O	PEDOT:PSS Graphene *for electrode*	√	√	x	x	182 × 10^3^	All-in-gel	281.2 F g^−1^ @0.1 A g^−1^	[55]
	Pluronic *MW 2800 Da*	H_2_SO_4_/H_2_O		x	x	x	√	3.9 (sol) 0.027 (gel)	PPy@carbon paper AC@carbon paper	110 F g^−1^@RT 10 F g^−1^@70 °C (*at 10 mV s^−1^*)	[57]
	PNIPAM-co-NMAM	0.5 M LiTFSI/ H_2_O	TEMED	√	x	x	√	Not provided	PPy@CC	168 mF cm^−2^@RT 34 mF cm^−2^@70 °C @0.5 mA cm^−2^	[60]
	PVA	H_3_PO_4_ H_2_O		√	x	x	x	Not provided	MWCNTs (*µSC*)	5.5 F cm^−3^ @ 10 mV s^−1^ (*total volume of electrodes*)	[94]
	PAA/PAM	1 M LiClO_4_ H_2_O	0.1 M Fe^3+^	√	√	x	x	40.1	AC	178.5 F g^−1^ @ 0.25 A g^−1^	[111]
	PVA/ PHEAA	NaCl H_2_O		√	√	x	x	1300	MWCNTs	2.89 mF cm^−2^ @0.05 mA cm^−2^	[113]
	PAM/ Lignin	6 M KOH H_2_O	MBAA Fe^3+^	√	√	√	x	345@RT 44@−40 °C	AC	210 F g^−1^@RT 176 F g^−1^@−40 °C @1 A g^−1^	[109]
	poly(AMPS-co-DMAAm)	H_2_O	Laponite GO	√	√	√	x	5	CNT/PANI	180 F g^−1^ @10 mV s^−1^	[110]
	PVA/PEO	H_2_SO_4_ H_2_O	GA	√	√	x	x	67.1	PPy-co-PANI	773 mF cm^−2^ @0.2 mA cm^−2^	[112]
	Agarose/ PAM	Li_2_SO_4_ H_2_O	MBAA	√	√	x	x	41	AC	84.7 F g^−1^ @0.2 A g^−1^	[117]
	Gelatin/ PHEAA	HCl H_2_O	Chitosan Na_2_MoO_4_	√	√	x	x	300	AC	84 mF cm^−2^ @1 mA cm^−2^	[118]
	PAO-g-PSBMA	7 M LiCl H_2_O	AGE	√	√	x	x	29.8 × 10^3^ @RT 3.4 × 10^3^ @−30 °C	m_S_Ti_3_C_2_T_x_ MXene	519 F cm^−3^@RT 317 F cm^−3^@−30 °C @1 A g^−1^	[126]
	PVA	LiCl EG/H_2_O		√	√	x	x	22@RT 2.38@−40 °C	CNTs	16 mF cm^−2^@RT 11 mF cm^−2^@−40 °C @1 mA cm^−2^	[127]
	PVA	H_2_SO_4_ EG/H_2_O	ARS	√	√	x	x	25.2@RT 8.5@−37 °C	AC	345 F g^−1^@RT 240 F g^−1^@−37 °C @0.5 A g^−1^	[128]
	PVA	H_2_SO_4_ DMSO/H_2_O	Graphene	√	√	x	x	390@RT 100@−65 °C	PANI	261.2 F g^−1^@RT 225 F g^−1^@−65 °C @0.33 A g^−1^	[129]
	HPC/PVA	LiClO_4_ Glycerol/ H_2_O		√	√	x	x	27.3@RT 5.7@−40 °C	AC	194.7 F g^−1^@RT 143.6 F g^−1^@−40 °C @2 A g^−1^	[130]
	PVA/ Alginate	Glycerol/ H_2_O	CaCl_2_	√	√	x	x	169@RT 135@−20 °C	PANI	141 mF cm^−2^@RT 129 mF cm^−2^ @−20 °C @1 mA cm^−2^	[131]
	PVA/ PHEAA	LiCl H_2_O	EGINA	√	√	x	x	~500@RT ~150@−40 °C	CC	20 mF cm^−2^@RT 8.1 mF cm^−2^ @−40 °C @5 mA cm^−2^	[133]
	Poly NIPAM/ PAM	LiOTf H_2_O	Bis[C_6_VI^+^][Br^−^]	√	√	x	√	1.75@RT 0.1@60 °C	MnO_2_	100 F g^−1^@RT 0.03 F g^−1^@60 °C @5 mV s^−1^	[135]
	Poly NIPAM	H_2_O	MBAA	√	√	x	√	Not provided	PPy NTs All-in-one *And* rGO/CNT	360 F g^−1^@RT 35 F g^−1^@40 °C @100 mV s^−1^	[137]
	PVA	H_3_PO_4_ H_2_O	TiO_2_	√	x	x	x	64.9	Graphene	69.2 F g^−1^ @20 mV s^−1^	[175]
	PAA	H_3_PO_4_ H_2_O	Vinyl-SiO_2_ NPs	√	√	√	x	8	PPy@CNTs	220 F g^−1^ @5 mV s^−1^	[198]
	PVA	H_2_SO_4_ H_2_O	PANI	√	√	x	x	Not provided *R = 2.2 Ω*	PVA/PANI *All-in-one*	300 mF cm^−2^ @2 mV s^−1^	[201]
	PVA	H_2_SO_4_ H_2_O	PANI	√	√	x	x	0.12	PVA/PANI *All-in-one* AC	1.88 F cm^−2^ @0.1 mA g^−1^	[202]
	PAAM	H_2_O	MBAA MXene/PANI 0.3 M Fe^3+^ PA	√	√	x	x	Not provided *R = 50.9 Ω* *@−20 °C* *R = 20Ω @RT*	MXene/PANI/PAAM *All-in-one*	645 mF cm^−2^@RT 355 mF cm^−2^ @−20 °C @5 mA g^−1^	[203]
	PAM	1 M LiCl EG/H_2_O	AgNW@Ag-CNTs BACA-Au NPs	√	√	√	x	~965 × 10^3^@ RT to −35 °C Not provided	AgNW@Ag-CNTs/PAM *All-in-one*	~168 mF cm^−2^ @RT to −35 °C @0.5 mA g^−1^	[204]
**Organogel**											
**BATTERIES**	PEG-UPy/PEO/PVdF-HFP	Liquid electrolyte *Not precise*		√	√	√	x	0.75	LiFePO_4_ Li metal	147.1 mA h g^−1^ @1 C	[34]
	PEGDA-UPy	1 M LiPF_6_ /EC/DMC	PEGDA Dipenta-erythritol penta-/hexa-acrylate	x	x	√	x	0.5	Li metal	150 mA h g^−1^ @0.5 C	[35]
	P(MMA-BA-(F-MA))	LiBOB EC:DMC (10 µL)	BMI	√	√	√	x	2.56	LiCoO_2_ Li metal	149 mA h g^−1^ @0.1 C	[36]
	DASHPE	1 M LiPF_6_ EC/DEC/DMC	BMI	√	√	√	x	0.16	LiFePO_4_ Li metal	133.1 mA h g^−1^ @0.1 C	[37]
	TF/TMI-based GPEs	LiTFSI (10 wt.%) EC/DEC		x	x	√	x	1.07	Li metal	140 mA h g^−1^ @0.2 C	[38]
	CNGPE	LiTFSI/ NMA	LiDFOB LiNO_3_ POSS-SH PDMS-DMA JDMA DSDA-DMA	√	x	√	x	0.77	LiFePO_4_ Li metal	150 mA h g^−1^ @0.5 C (118.3 mA h g^−1^ @0.1 C for pouch cell)	[40]
	IBshPE	0.3 M LiDFOB/ 0.02 M LiPF_6_/ 0.8 M LiTFSI FEC/EC	NH_2_-PEG-NH_2_ 2-FBA	√	√	√	x	5.08	LiFePO_4_ Li metal	156.6 mA h g^−1^ @0.1 C	[42]
	PBPE	0.3 M LiDFOB/ 0.02 M LiPF_6_/ 0.8 M LiTFSI	NH_2_-PEG-NH_2_ BTC	√	√	√	x	4.79	LiFePO_4_ Li metal	157.8 mA h g^−1^ @0.1 C	[43]
	PETPTA	0.2 M LiDFOB/ 1 M LiTFSI/ FEC/FEPE		√	x	x	√	1.19	Li metal	220 mA h g^−1^ @ 0.1 C	[46]
	PPEGMA *on Whatman substrate*	NaTFSI	TEP FEC	√	x	x	√	0.91	Na_3_V_2_(PO_4_)_3_ Na metal	117.2 mA h g^−1^ @0.1 C	[47]
	PEGDA	LiTFSI	PEGDA TEP TiO_2_ NPs	x	x	x	√	0.07	LiFePO_4_ Li metal	132.3 mA h g^−1^ @0.2 C	[48]
	PAN@ Paraffin *Electrospun*	1 M LiPF_6_ EC/DEC/DMC		x	x	x	√	1.4	LiFePO_4_ Li metal	150 mA h g^−1^ @0.2 C	[53]
	PFE/PEGA	1 M LiBOB LiNO_3_ (3 wt.% of polymer) EC/DMC	VC	x	x	x	√	2.28@RT 0.14@−10 °C	LiFePO_4_ Li metal	170 mA h g^−1^ @0.1 C	[59]
	MOF/AMImTFSI	LiTFSI		x	x	x	x	2.3	Li metal Si anode	3250 mA h g^−1^ @0.1 C	[68]
	PMMA/PAN	1 M LiTFSI TEGDME	ETPTA	√	x	x	x	0.33	LiFePO_4_ Li metal	156.8 mA h g^−1^ @0.2 C	[75]
	Gelatin	Li_2_SO_4_ H_2_O	ZnSO_4_	√	√	x	x	61	LiMn_2_O_4_ Zn metal	110 mA h g^−1^ @25 mA g^−1^	[138]
	PANa	6 M KOH H_2_O	0.2 Zn^2+^	√	√	x	x	126@RT 163@50 °C 57@−20 °C	NiCo Zn metal	110 mA h g^−1^ @−20 °C@9 C 157 mA h g^−1^ @50 °C@8 C	[139]
	PANa	6M KOH H_2_O	0.2 M Zn^2+^	√	√	x	x	200	NiCo Zn metal	87 mA h g^−1^ @11.3 C	[140]
	PANa	6M KOH H_2_O	0.2 M Zn^2+^	√	√	x	x	170	NiCo@CC Zn@CC	259 mA h g^−1^ @5.8 C	[141]
	Xanthan gum	H_2_O	4 M Zn^2+^	√	x	x	x	12.08@RT 2.54@−20 °C	NH_4_V_3_O_8_. 1.9H_2_O Zn metal	426 mA h g^−1^@RT 201 mA h g^−1^ @−20 °C @0.2 A g^−1^	[142]
	PAAm	H_2_O	MBAA 1 M Zn^2+^ 1 M Mn^2+^	√	√	x	x	Not provided (*R = 36.8 Ω*)	MnO_2_ Zn metal	230.5 mA h g^−1^ @1 C	[143]
	PAAm/ Alginate	H_2_O	2 M Zn^2+^ 0.1 M Mn^2+^	√	√	x	x	43.2	MnO_2_ Zn metal	300.4 mA h g^−1^ @0.11 A g^−1^	[144]
	PAM/ Alginate	H_2_O	MBAA 2 M Zn^2+^ 1 M Na^+^	√	√	x	x	19.74	Prussian blue@CNTs Zn metal	136.4 mA h g^−1^ @0.1 C	[145]
	PVdF	1 M LiPF_6_ EC/DMC		√	x	x	x	0.5	LiFePO_4_ Graphite	150 mA h g^−1^ @C/7	[147]
	P(MA-co-AN)/PVA	1 M LiPF_6_ EC/DEC/DMC	MBAA	√	√	x	x	0.98	LiCoO_2_ Graphite	140 mA h g^−1^ @0.1 C	[148]
	GECO	1 M LiTFSI DOL/DME	ETU MgO	√	√	x	x	0.24	Carbon—sulfur cathode Li metal	700 mA h g_Sulfur_^−1^ @0.1 C	[149]
	CMNC	1 M LiPF_6_ EC/DEC/ DMC	Epichlorohydrin	√	√	x	x	3.93	NMC532 Li metal	151.4 mA h g^−1^ @0.2 C	[150]
	Allyl-modified cellulose	1 M LiPF_6_ EC/ DMC		√	√	x	x	4.36	LiFePO_4_ Li metal	150.6 mA h g^−1^ @0.2 C	[151]
	PVdF-HFP	0.5 M NaPF_6_ TMP		√	x	x	√	1.4	Na battery Red P/NaHg SC AC	225 mA h g^−1^ @31 mA g^−1^ 100 F g^−1^ @10 mV s^−1^	[152]
	PEI	1 M LiTFSI DOL/DME	PEGDE	√	x	x	x	0.75	Carbon–sulfur cathode Li metal	1100 mA h g_Sulfur_^−1^ @0.1 C	[153]
	PEGMEA	1 M LiPF_6_ EC/DEC	ETPTA	√	√	x	x	1.66	LiCoO_2_ Li metal	140 mA h g^−1^ @0.1 C	[154]
	PDEI/ PVdF-HFP	1 M LiPF_6_ EC/DMC		√	√	x	√	1.78	LiFePO_4_ Li metal	155.5 mA h g^−1^ @0.1 C	[157]
	P(AN-DEVP)	1 M LiPF6 EC/DEC/DMC		√	x	x	√	1.4	LiFePO_4_ Li metal	145.4 mA h g^−1^ @0.5 C	[158]
	Poly(VDF-co-VEPFSIS)	EC/PC	*Nota bene* *Single ion GPE*	√	x	x	x	0.5	NMC Li metal	143 mA h g^−1^ @0.05 C	[165]
	PI-LiCPSI/ PVdF-HFP	EC/DMC	*Nota bene* *Single ion GPE*	√	x	x	x	0.14	LiFePO_4_ Li metal	141 mA h g^−1^ @0.2 C	[166]
	PE-co-PVA-graft-LiCPSI PVdF-HFP	EC/DMC	*Nota bene* *Single ion GPE*	√	x	x	x	0.057	LiFePO_4_ Li metal	100 mA h g^−1^ @1 C	[167]
	P(MPEGA-AMPSLi)	EC/DMC	*Nota bene* *Single ion GPE*	√	x	x	x	0.028	LiFePO_4_ Li metal	146.2 mA h g^−1^ @0.2 C	[168]
	P(LiSTFSI)	DME/DOL	PEGDMA *Nota bene* *Single ion GPE*	√	x	x	x	0.027	LiFePO_4_ Li metal	81.1 mA h g^−1^ @0.1 C	[169]
	PVdF-HFP /PEO	1 M LiTFSI EC/DMC	SiO_2_ NPs	√	√	x	x	1.12	LiFePO_4_ Li metal	152.5 mA h g^−1^ @0.1 C	[170]
	PVAc/ PVdF-HFP	1 M LiPF_6_ EC/EMC/ DMC	SiO_2_ NPs	√	x	x	x	1.3	NMC532 C µbeads	159.8 mA h g^−1^ @0.5 C	[173]
	PTC	1 M LiPF_6_ EC/DEC	Graphene oxide	√	x	x	x	2.28	LiCoO_2_ Li_4_Ti_5_O_12_	143 mA h g^−1^ @1 C	[184]
	PTC	1 M LiPF_6_ EC/DEC	Graphene oxide	√	x	x	x	5.5	LiCoO_2_ Graphite	161.3 mA h g^−1^ @0.2 C	[185]
	PVdF-HFP /PEG	LiTFSI	LATP	√	x	x	x	0.81@40 °C	LiFePO_4_ Li metal	144.8 mA h g^−1^ @0.1 C	[186]
	PTC	LiTFSI 1 M LiPF_6_ EC/DEC	LLZTO	√	x	x	x	1.8	LiCoO_2_ Li_4_Ti_5_O_12_	151.9 mA h g^−1^ @0.2 C	[187]
	PVA		Glutaraldehyde LLZO	√	x	x	x	0.09	LiFePO_4_ Li metal	158 mA h g^−1^ @0.1 C	[189]
	PVdF-HFP P(MMA-co-HEMA)	1 M LiClO_4_ /PC	SiO_2_	√	x	x	x	2.63	LiMn_2_O_4_ Li metal	139.6 mA h g^−1^ @0.1 C	[190]
	PV-CD/ BSIP		SiO_2_ Li-MMT PEGDA	√	√	x	x	0.25	LiFePO_4_ Li metal	~150 mA h g^−1^ @0.2 C	[192]
	Fabric/TPU	LiTFSI G4	SiO_2_ aerogel	√	√	x	√	1.02	MWCNTs Li metal	930 mA h g^−1^ @0.5 A g^−1^	[193]
**SUPERCAPACITORS**	PVdF-HFP	NaPF_6_ TMP		√	x	x	√	1.4	AC	100 F g^−1^@10 mV s^−1^	[152]
	PVdF-HFP	ACN/MF PC	[EMI^+^][BF_4_^−^]	√	√	x	x	9.21@RT 2.95@−60 °C	AC	120.4 F g^−1^@RT 118.9 F g^−1^@−60 °C @0.5 A g^−1^	[160]
	PVA	Trimesic acid DMSO	Phenylene diamine	√	√	x	x	45.45	AC	322.5 F g^−1^@0.1 A g^−1^	[162]
	PVA	LiCl EG	SiO_2_ STF	√	√	x	x	2.3@RT ~7@80 °C	AC	21F g^−1^@RT 40 F g^−1^@80 °C @1 A g^−1^	[200]

**Electrode materials: ZVO** = Zr_3_V_2_O_7_(OH)_2_.2H_2_O; **CC** = Carbon cloth; **AC** = activated carbon; **GO** = graphene oxide; **rGO** = reduced graphene oxide; **(MW)CNTs** = (multi-walled) carbon nanotubes; **NMC532** = LiNi_0.5_Mn_0.3_Co_0.2_O_2_; **PANI** = polyaniline; **PPy** = polypyrrole; **PEDOT:PSS** = Poly(3,4-ethylenedioxythiophene) polystyrene sulfonate; **Red P** = Red phosphorous; **3DMC** = 3D interconnected mesoporous carbon. **Electrolyte: EG** = ethylene glycol; **DMSO** = Dimethyl sulfoxide; **LiOTf** = Lithium triflate; **Bis[C_6_VI^+^][Br^−^]** = 4,4′-(dodecane−1,12-diyl)bis-(1-vinyl−4H-imidazolium)bromide; **EC** = ethylene carbonate; **FEC** = fluoroethylene carbonate; **DMC** = dimethylcarbonate; **LiBOB** = lithium bis(oxalato)borate; **LiDFOB** = lithium difluoro(oxalato)borate; **DEC** = diethylcarbonate; **NMA** = N-methylacetamide; **FEPE** = 1,1,2,2-tetrafluoroethyl−2,2,3,3-tetrafluoropropyl ether; **TEP** = triethyl phosphate; **TMP** = Trimethyl phosphate; **DME** = dimethyl ether; **TEGDME** = tetraethlene glycol dimethyl ether; **DOL** = 1,3 dioxolane; **G4** = tetraglyme; **EMI** =1-ethyl−3-methylimidazolium; **TFSI** = bis(trifluoromethane)sulfonamide; **DCA** = Dicyanamide; **ChCl** = Choline chloride; **MUBI-TFSI** = 1-[(2-methacryloyloxy)undecyl]−3-butylimidazolium bis(trifluoromethane)sulfonamide; **FAP** = tris(pentafluoroethyl) trifluorophosphate. **Monomers/Polymer networks: PANa** = poly(sodium acrylate); **CBH** = Cellulose-based hydrogels; **PEO** = polyethylene oxide; **PEG** = polyethylene glycol; **PPO** = polypropylene oxide; **Pluronic** = PEO-PPO-PEO triblock; **PEGA** = polyethylene oxide acrylate; **PEGDA** = polyethylene oxide diacrylate; **PEGDM** = polyethylene oxide dimethacrylate; **NIPAM** = N-isopropylacrylamide; **AM or AAm** = acrylamide; **P(ICZn-AAm)**-IC = iota carrageenan; **NFC** = Nanofibrillated cellulose; **HPC** = Hydroxypropyl cellulose; **CMNC** = carboxymethylated nanocellulose; **PVA** = poly(vinyl alcohol); **NMAM** = N-methylol acrylamide; **AMPS** = 2-Acrylamido−2-methylpropane sulfonic acid; **DMAAm** = N,N-dimethylacrylamide; **PAA** = Polyacrylic acid; **PHEAA** = poly(hydroxyethylacrylamide); **PAO** = poly(amidoxime); **PSBMA** = Poly([2-(methacryloyloxy)ethyl]dimethyl-(3-sulfopropyl)ammonium hydroxide); **AGE** = allyl glycidyl ether; **UPy** = ureido-pyrimidione; **DASHPE** = thermally reversible self-healing gel polymer electrolyte; **CNGPE** = three-dimensional (3D) cross-linked network gel polymer electrolyte; **PDMS-DMA** = bis(3-aminopropyl)-terminated poly(dimethylsiloxane) dimethacrylate; **PETPTA** = poly(ethoxylated trimethylolpropane triacrylate); **PAN** = polyacrylonitrile; **PFE** = 2,2,3,3,3-pentafluoropropyl acrylate; **PMMA** = polymethyl methacrylate; **PVdF-HFP** = Polyvinylidene fluoride-Hexafluoropropylene; **GECO** = Poly(epichlorohydrin) rubber with x wt.% chlorine; **PEI** = polyethylenimine; **PDEI** = poly(1,2-diethoxyethylimidazolium); **DEVP** = vinylphosphonate; **VEPFSIS** = perfluoro−2-methyl−3-oxa−5-sulfonimido[−3-oxa−5-sulfonyl fluoride] vinyl ether; **PTC** = poly(vinylidene fluoride-tri-fluoroethylene-chlorofluoroethylene); **PHEMA** = Poly(2-hydroxyethyl methacrylate); **TPU** = thermoplastic polyurethane; **CA** = cellulose acetate; **MPBI** = 1-[(2methacryloyloxy) propyl]−3-butylimidazolium; **NBR** = nitrile butadiene rubber; **PMT** = poly[2-(methacryloy-loxy)ethyl]trimethylammonium bis(trifluoromethanesulfonyl) imide; **PES** = poly[1-ethyl−3-methylimidazolium (3-sulfopropyl)methacrylate]. **Crosslinkers/Others: DVS** = divinyl sulfone; **MBAA** = N,N′-methylenebisacrylamide; **TEMED** = N,N,N′,N′-Tetramethylethylenediamine; **ARS** = Alizarin Red-S; **POSS-SH** = thiol-functionalized polyhedral oligomeric silsesquioxane; **DSDA-DMA** = ((((disulfanediylbis(4,1-phenylene))bis(azanediyl))bis (carbonyl))bis(azanediyl))bis(ethane−2,1-diyl) bis(2-methylacrylate); **JDMA** = Jeffamine dimethacrylate; **2-FBA** = 2-Formylphenylboronic acid; **BTC** = benzene−1,3,5-tricarbaldehyde; **V-CD** = vinyl modified gamma cyclodextrine; **MOF** = metal organic framework; **ETPTA** = ethoxylated trimethylolpropane triacrylate; **ETU** = ethylene thiourea; **LATP** = Li_1.3_Al_0.3_Ti_1.7_P_3_O_12_; **LLZTO** = Li_6.4_La_3_Zr_1.4_Ta_0.6_O_12_; **LLZO** = Li_7_La_3_Zr_2_O_12_; **HPDS** = bis(4-hydroxyphenyl) disulfide; **IPDI** = alicyclic isophorone di-isocyanate; **Li-MMT** = Li-based montmorillonite; **TPB** = triphenylbenzene; **DMTP** = dimethoxyterephthaldehyde; **COF** = covalent organic framework; **PEGDE** = Poly(ethylene glycol) diglycidyl ether; **DADMA** = diallyldimethylammonium; **TEOS** = Tetraethylorthosilicate; **EGINA** = 4,9-dioxo−5,8-dioxa−3,10-diazadodecane−1,12-diyl diacrylate; **GA** = glutaraldehyde; **PA** = phytic acid.

**Table 2:** Cost of typical chemicals for GPE synthesis.

Item	Cost	Item	Cost
Acrylic acid	USD ~1500 per ton	EMITFSI	USD ~50,000 per ton
MBAA	USD ~37,000 per ton	NaOH	USD ~500 per ton
Ethylene glycol	USD ~580 per ton	Potassium persulfate	USD ~2000 per ton
PEO	USD 1200–1800 per ton	PEGDM	USD ~30,000 per ton
Pluronic	USD ~4000 per ton		
Cellulose	USD 2000–5000 per ton		
